# Marine Biotoxins as Potential Nematocide: Impact of *Cassiopea andromeda* Venom Against Acute and Chronic Trichinellosis in Murine model

**DOI:** 10.1007/s11686-026-01256-7

**Published:** 2026-04-06

**Authors:** Rasha A. Elmahy, Amina M. Salama, Nahla A. Radwan, Alaa Y. Moustafa, Samar F. Harras, Mona M. Elwan

**Affiliations:** 1 Zoology Department, Faculty of Science, Tanta University, Tanta, Egypt; 2 Medical Parasitology Department, Faculty of Medicine, Tanta University, Tanta, Egypt; 3 Zoology Department, Faculty of Science, Sohag University, Sohag, Egypt

**Keywords:** *Cassiopea andromeda*, *Trichinella spiralis*, Venom, Albendazole, SEM, Oxidative stress, Immunohistochemical analysis, Histopathological alterations

## Abstract

**Background:**

A significant challenge in the treatment of trichinellosis is addressing theencysted larvae. The efficacy of albendazole on larvae appears to be limited, underscoring theurgent necessity for developing effective alternatives. Recently, marine biotoxins have beeninvestigated as possible substitutes for traditional medications. In the present work, we assessedthe nematocidal potential of the jellyfish Cassiopea andromeda’s venom against the enteral andparenteral phases of Trichinella spiralis in mice.

**Methods:**

A total of 115 BALB/c mice werecategorized into two phases: acute and chronic. Each was divided into five groups: uninfecteduntreated group, untreated group infected with T. spiralis , uninfected group and treated withjellyfish venom, infected group and administered albendazole, and infected group and treatedwith jellyfish venom. In vitro study exposed adults and larvae of T. spiralis to the Lethalconcentration (LC90) of C. andromeda venom, and ultrastructural changes were observed. Invivo assay included haematological and biochemical analyses. Histopathological andimmunohistochemical assessments evaluated the host tissue’s alterations. ANOVA and post hoctests were used in the statistical analysis.

**Results:**

The present results showed that the LC90 ofthe venom caused complete mortality of adults. The infected and venom-treated group of micegained weight, achieved a 85.23% reduction in adult worms, and showed verified structural improvement, diminished degenerative changes, and decreased P53 expression. High levels of oxidative stress indicators and liver enzymes were observed.

**Conclusions:**

Venom treatmentdecreased inflammation and restored antioxidant levels. C. andromeda venom may be aninteresting alternative for treating T. spiralis' intestinal and muscle phases; however, furtherresearch is needed to clarify its structure, production, molecular targets, and methods ofadministration.

**Supplementary Information:**

The online version contains supplementary material available at 10.1007/s11686-026-01256-7.

## Introduction

*Trichinella* causes the worldwide foodborne parasitic disease trichinellosis, which poses a public health threat to humans and causes significant economic losses in the pig industry [1]. Among the *Trichinella* species, *Trichinella spiralis* (*T. spiralis*) is the most pathogenic to humans [2], with approximately 10,000 new cases reported globally each year [3]. The disease is a re-emerging public health concern, with outbreaks having been reported in 55 countries [4, 5]. While the infection can affect various mammalian hosts, the primary risk factor for human infection is linked to cultural and dietary habits [1, 6].

It is known that *T. spiralis* completes its life cycle in the same host and that the adult worms and larvae occupy distinct intracellular niches. These are two distinctive traits that affect the host’s immunological response. Larvae live in skeletal muscle fibers, whereas adults invade the intestinal epithelium [7]. Adult worms trigger acute inflammatory responses, leading to various intestinal pathological changes [8]. They are ejected from the intestine within 17 days post-infection, making the intestinal phase critical in determining the disease’s progression [9]. Muscle infection is accompanied by significant inflammation, which is an important factor contributing to morbidity and mortality [10].

During infection, a strong Th2 cell response triggers a transient inflammatory response that eventually results in the worms’ expulsion [11]. Soluble mediators such as IL-4, IL-5, IL-9, IL-13, histamine (released from mast cells), and antibodies (IgE and IgG1) are all part of this Th2-driven immune response, which aids in the expulsion of adult worms. Even though these Th2 cytokines are essential for this process, the precise pathways and mechanisms remain unknown [12]. *T. spiralis* excretory-secretory (ES) products stimulate the immune system over time, activating regulatory components in the process.

In some cases, the death of parasites due to anthelmintic treatment can worsen inflammatory reactions, especially in dynamic organs like the brain or heart. Thus, anti-inflammatory medications are often necessary to manage the infection [13]. However, both non-steroidal and steroidal anti-inflammatory agents have potential side effects and contraindications that limit their use [14, 15]. Anthelmintics are most effective when given early, during the larvae’s migration to the muscles or while adult worms are still in the small intestine. Most patients seek medical attention at later stages when larvae have already settled extensively in skeletal muscle fibers [1]. Traditional anthelmintic treatments, primarily benzimidazole derivatives like albendazole (ABZ) and mebendazole (MBZ), have limited effectiveness against encysted larvae [16, 17]. Another complication is the drugs’ limited bioavailability, due to their poor solubility and low water solubility, which hinders their effectiveness. Additionally, these drugs have faced resistance issues, are contraindicated for young children and pregnant women [18, 19]. As such, albendazole remains a key drug, but its limitations necessitate the exploration of alternative or complementary therapies, such as antioxidants and natural compounds, to enhance therapeutic outcomes [20, 21]. Recent research has also suggested that combining anti-trichinellosis medications, which can modulate inflammation and reduce larval encapsulation, could have synergistic effects, ultimately aiming to reduce muscle damage [20].

Natural products could be used in addition to or as a substitute for chemical drugs to treat parasites in humans and animals. This has resulted in an ongoing search for safer and more effective treatments, particularly those derived from natural sources [22, 23].

For more than five years, drug discovery efforts have increasingly focused on unconventional sources, such as marine toxins (including those from cnidarians, molluscs, sponges, and sea cucumbers), in the hopes of finding more effective therapeutic agents with novel chemical structures and distinct mechanisms of action [24, 25].

The vast chemical diversity of marine products offers an extensive resource for discovering novel bioactive molecules for pharmacological applications. Jellyfish, members of the phylum Cnidaria, are one of the marine organisms that have contributed pharmacologically active compounds (enzymes, peptides, hemolysin, cytolysin, and neurotoxin), which possess antimicrobial, antioxidant, wound**-**healing, and cytotoxic properties. Additionally, the antitumor activity and growth-inhibitory effects of jellyfish venom have been previously studied [26–29]. In general, these components can act as antigens in humans, triggering an immune response that includes the production of specific antibodies and the activation of immunological memory. This feature makes jellyfish venom a topic of interest not only for therapeutic applications but also for understanding immune responses [30–32].

However, there has been a recent surge in interest regarding how jellyfish venom works against various nematodes. Elmahy et al. [33] were the first to apply the venom of *C. andromeda* against *Toxocara canis* third**-**stage larvae and discovered a unique impact. The present work aims to assess the therapeutic potential of *C. andromeda* venom against both the intestinal (acute) and muscular (chronic) phases of *Trichinella spiralis* infection by comparing its efficacy to the reference drug albendazole through a parasitological and pathological assessment.

### Materials and Methods

**Fig. 1:**
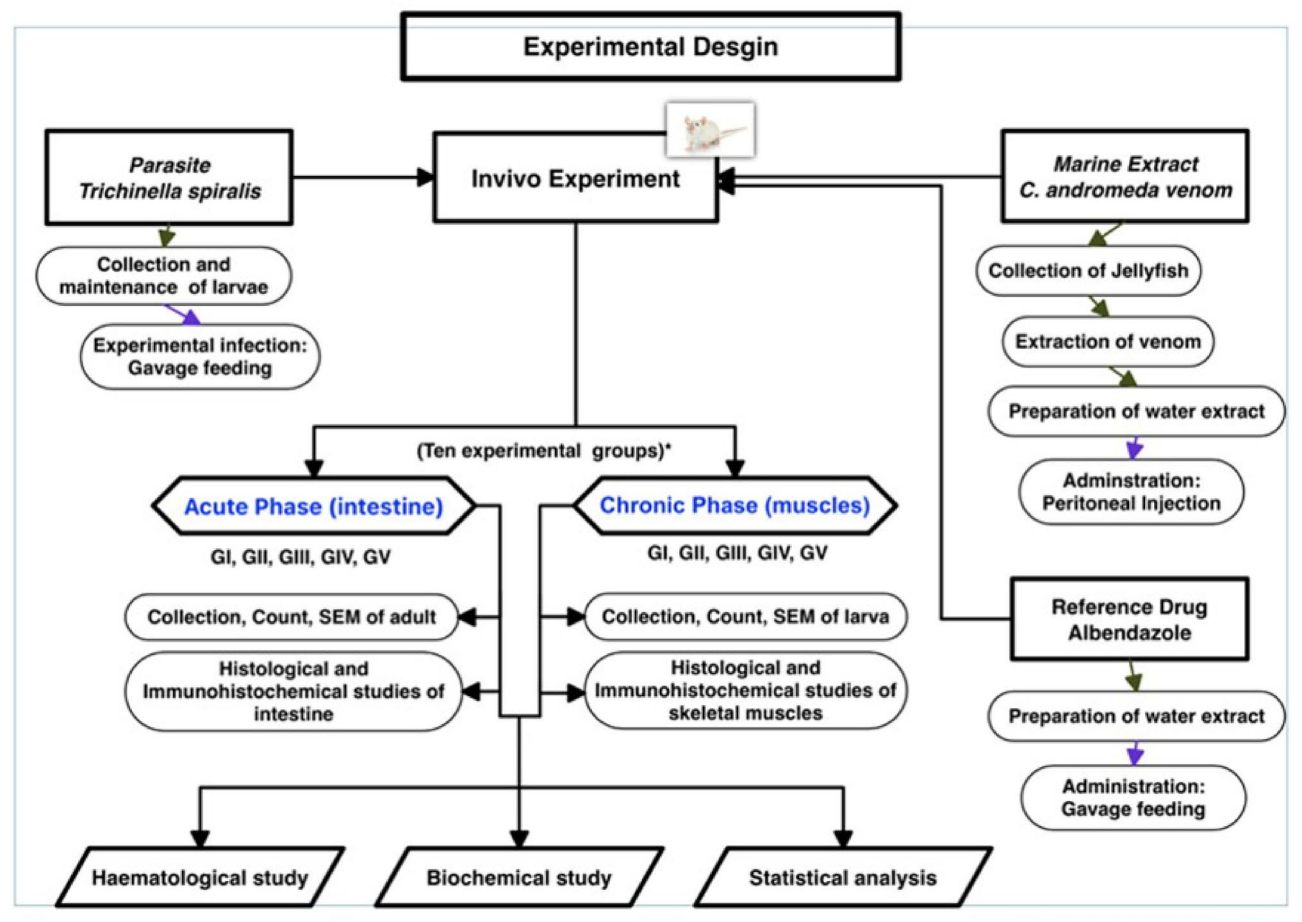
A flow chart illustrating the procedures of the present experimental study

The study’s plan is illustrated in Fig. 1.

### Animals

A total of 115 male BALB/c mice (6–8 weeks old, 25–30 g in weight) were obtained from the Theodore Bilharz Research Institute in Giza, Egypt. They were kept in standard cages at 25 ± 2 °C, with access to standard feed and water and were acclimatized for 7 days before the experiment.

### Parasite

A *T. spiralis* strain, isolated from infected pork obtained from a Cairo slaughterhouse, was identified and genotyped as *T. spiralis* (ISS6158) by the European Union Reference Laboratory for Parasites at the Superior Institute of Health in Rome, Italy. It was successfully maintained in mice at the Medical Parasitology Department Laboratory, Faculty of Medicine, Tanta University.

Infected mice were sacrificed, skinned, and eviscerated. Unnecessary parts, such as teeth, ears, and tails, were removed before the carcasses were cut into small pieces and minced. The minced tissue was digested in synthetic gastric juice (1% pepsin w/v and 1% concentrated HCl v/v in warm distilled water) at 37 °C for two hours with continuous stirring to release larvae. The digest was sieved through a 50-mesh copper sieve lined with gauze, followed by a finer 200-mesh sieve to collect the larvae, which were subsequently washed several times with distilled water and allowed to sediment.

The supernatant was removed, and larvae in the sediment were counted under a microscope using a McMaster chamber [34].

### Jellyfish Venom

Mature specimens of the jellyfish *C. andromeda* were collected from the Egyptian coast of the Red Sea, 17 km south of the city of Safaga, Red Sea Governorate. The extraction of the venom was according to Burnett and Long [35] and Mustafa et al. [36] with some modification. Nematocysts and tentacles (about 50 gram) were separated and stored immediately in seawater, 20 gm of tissues was homogenized and kept at 4 °C for 2 days, and then centrifuged at 12,000 rpm for 15 min at 4 °C. The supernatant was freeze-dried and stored at − 80 °C until needed.

### Albendazole

Albendazole: @Alzental oral suspension (100 mg/5 ml), manufactured by Eipico Pharmaceutical Company, Egypt.

### Preparing the Experimental Groups

Severely infected mice were slaughtered and artificially digested using the HCl-pepsin artificial digestion method [37] to collect larvae.

One hundred **-** fifteen male mice were selected for the experimental work and divided into ten groups. Seventy-five mice were infected with 200 viable *T. spiralis* larvae per mouse, administered via gavage [38]. The remaining forty mice were left uninfected as controls.

The study was structured to investigate the impact of *C. andromeda* venom on mice infected with *T. spiralis*, and was divided into two distinct experimental phases: the intestinal (acute) phase assessed on the 7th day post-infection (dpi) and the muscular (chronic) phase assessed on the 36th day post-infection (pi).

The intestinal (acute) phase of the infection comprised five groups, each with 10 mice: G I, uninfected and untreated (control group); G II, infected with *T. spiralis* and untreated; G III, uninfected and treated with jellyfish venom; G IV, infected with *T. spiralis* and administered albendazole; and G V, infected with *T. spiralis* and treated with jellyfish venom.

The muscular (chronic) phase of the infection comprised five groups, each with 13 mice: G I, uninfected and untreated (control group); G II, infected with *T. spiralis* and untreated; G III, uninfected and treated with jellyfish venom; G IV, infected with *T. spiralis* and treated with albendazole; and G V, infected with *T. spiralis* and treated with jellyfish venom.

### In Vitro Study

On the 7th day pi (the acute phase), the intestines of untreated infected mice (G II) were dissected and opened longitudinally. Adult male and female *T. spiralis* were collected promptly. On the 36th day pi (the chronic phase), mice from G II were euthanized, dissected, and their muscles were sliced into small fragments and digested [39]. The larvae were obtained using the pepsin digestion method [37]. The in vitro experiment was conducted promptly after collecting the adults and larvae at room temperature, avoiding the routine incubation at 37 °C.

An in vitro investigation into the effect of a sub-lethal dose (LC90) of *C. andromeda* venom water extract [33] was performed in a laminar flow cabinet (Nuaire NU 437-400E) under a normal gas phase at 37^◦^C. Twenty adult worms and twenty larvae were incubated separately in RPMI 1640 medium (with stable glutamine) and exposed for 10 h to the LC90 of *C. andromeda* venom. The control group, incubated in RPMI medium only, and the albendazole group, treated with 250 µg/mL of albendazole [40], were included to serve as comparative controls for the examination results. After incubation, adults and larvae from the three groups were washed and immediately fixed in 4% buffered-phosphate glutaraldehyde (GA) solution (pH 7.4) at 4 °C for scanning electron microscopy investigations.

### Scanning Electron Microscopy of Adults and Larvae of *T. spiralis*

Fixed specimens were post-fixed in 1% osmium tetroxide, dehydrated through a consecutive series of alcohol, dried to the critical point with carbon dioxide, and coated with gold **-** platinum. Samples were observed and photographed with a JSM‐IT 200 JEOL scanning electron microscope at an accelerating voltage between 15 and 20 keV in the Electron Microscopy Unit at Alexandria University, Egypt.

###  In Vivo Study and Drug Administration

In the acute phase, mice in G III and G V received daily intraperitoneal injections of 0.4–0.7 ml of a water extract of jellyfish venom toxin prepared at a concentration of 44 µg/ml [33], starting from day 2 and continuing until day 6. Conversely, mice in G III and G V in the chronic phase received the same treatment regimen, with daily injections of the same dose, beginning on day 28 and concluding on day 35.

The water solution of albendazole was prepared as a white suspension of 100 mg/5 ml and was administered orally at a dosage of 100 mg/kg once daily for five consecutive days [41] to mice in G IV in the acute phase, starting from day 2 and continuing until day 6. Conversely, mice in G IV in the chronic phase received the same treatment regimen with the same dose, beginning on day 28 and concluding on day 35.

Mice were euthanized using Isoflurane, followed by cervical dislocation to confirm death on days 7 (acute phase) and 36 dpi (chronic phase).

#### Alteration of the Host Body Weight

The alteration is considered an indicator of disease impact and therapeutic efficiency. The initial body weight (initial b.wt) and final body weight (final b.wt) of all mice were measured. The following formula was used to determine the percentage change in body weight:

$$ \begin{aligned} \% {\text{ change = }} & \left[ {\left( {{\text{final b}}.{\text{wt }} - {\text{ initial b}}.{\mathrm{wt}}} \right)/{\text{initial b}}.{\mathrm{wt}}} \right] \\ \quad & \times {\text{ 1}}00 \\ \end{aligned} $$  

#### Total Count of Adults and Larvae of *T. spiralis*

On the 7th dpi, mice from G II, G III, G IV, and G V were fasted overnight, anesthetized with 4–5% isoflurane in 100% oxygen, and dissected. Intestines were removed into separate Petri dishes, opened longitudinally in 0.9% normal saline, and cut into small pieces, roughly 1 cm in size, to isolate the adult worms. Samples were incubated at 37 °C in 100 ml of Hanks’ Balanced Salt Solution (HBSS) for 3 h. Next, the fluids were carefully pipetted, collected in tubes, and centrifuged at 1500 rpm for 5 min. After centrifugation, the supernatant was discarded, and the sediment was reconstituted in 3–5 drops of HBSS. Using a pipette, the samples were examined at a magnification of 20x [42].

On the 36th dpi, mice from G II, G III, G IV, and G V were euthanized and dissected, and their tissues were sliced into small fragments for digestion following the methodologies outlined by [39]. Larvae were extracted using the pepsin digestion method, with enumeration performed microscopically using a McMaster counting chamber (Faust, Germany). Larval burdens were quantified as the number of larvae per gram of carcass digested [43].

#### Haematological Investigations

Blood samples were collected at the time of euthanasia from mice in all study groups on the 7th and 36th dpi of the experiment; about 1–1.5 mL of blood was obtained from each mouse via the cervical vein. The examined parameters were red blood cell (RBC) count, white blood cell (WBC) count, haemoglobin (Hb) concentration (g/dL), haematocrit (Hct) value (%), and platelet (PLT) count.

#### Biochemical Analysis

##### Liver Function Biomarkers

The liver’s enzyme activities in all groups under study were assessed using the methods described by [44, 45]. Enzymes included serum alanine aminotransferase (ALT) and serum aspartate aminotransferase (AST). Total proteins were calculated according to [46].

##### Oxidative Stress Markers (CAT, SOD, GSH-Px and MDA)

One gram of each tissue type (acute tissue and skeletal muscle) was homogenised separately in an ice-cold 50 mmol/l potassium phosphate buffer solution (pH 7.4) containing KCl (1.15 g/100 ml) to make the tissue homogenate. Homogenisation was performed using a Cole-Parmer Instrument (USA) Ultrasonic Sonicator 4710. The homogenates were then cooled at 4 °C and centrifuged at 4000 rpm for 5 min. The recovered supernatant was stored at -80 °C.

Superoxide dismutase (SOD) activity was determined using an ELISA kit (Cat No. CSB-E08555r). The principle is based on measuring the inhibition of the reduction of p-nitro tetrazolium blue (NBT). Xanthine and xanthine oxidase were used to generate superoxide anion radicals, which react with 2-(4-iodophenyl)-3-(4-nitrophenol)-5-phenyltetrazolium chloride to quantitatively form a red formazan dye [47].

Catalase (CAT) activity in each sample was measured with an ELISA kit (Cat No. MBS2600683). Hydrogen peroxide (H₂O₂) breakdown is catalyzed by catalase, followed by the reaction of ammonium molybdate and excess H₂O₂. CAT activity is determined by the absorbance of the residual H₂O₂ at 450 nm [48].

Glutathione peroxidase (GSH-Px) activity was determined by an ELISA kit (Cat No. CSB-E12146r) based on the changes in the NADPH level. Hydrogen peroxide and cumene hydroperoxide (Sigma, St. Louis, MO) were used as substrates; they were reduced by GPX with glutathione as the reducing agent [49].

Malondialdehyde (MDA) level was determined using an ELISA kit (CAT# ab238537, Abcam), based on the reaction of MDA with thiobarbituric acid (TBA) under acidic conditions at approximately 95 °C, forming an MDA-TBA₂ adduct [50].

All data were measured using a spectrophotometer. The enzyme units (U per millilitre; total activity) were calculated as the change in absorbance per millilitre.

##### Interleukin-6 (IL-6) in Small Intestine and Skeletal Muscle Tissue Homogenates

The IL-6 level (Cat No. ab234570, Rat IL-6 SimpleStep ELISA^®^ Kit) was measured in both acute and skeletal muscle tissue homogenates using an automated microplate ELISA reader (Chemwell^®^ 2099, USA) at 450 nm with a correction wavelength of 630 nm, according to the manufacturer’s instructions.

#### Histopathological Assessment

After the dissection of mice, samples from the small intestine and skeletal muscle from all studied groups were immediately fixed in 10% formalin saline for 24 h. They were then dehydrated in an ascending series of alcohol, cleared in xylene, and embedded in paraffin wax at 56 °C for 24 h. Paraffin blocks were sectioned to a thickness of 5 μm and then processed following the protocol described in [51]. Finally, slides were examined using a light electric microscope (Olympus CX41, Japan).

#### Immunohistochemical Study

P53 immunohistochemical analyses were performed on paraffin sections of small intestine and skeletal muscle specimens using the Avidin-Biotin Complex (ABC) method [52]. Briefly, the sections were deparaffinized, hydrated, and treated with 3% H_2_O_2_ to eliminate endogenous peroxidase activity. The sections were then incubated with a P53 monoclonal antibody to detect apoptotic cells, followed by a biotin-conjugated secondary anti-mouse antibody and a streptavidin-conjugated peroxidase complex. The color reaction was developed using 3,3’-diaminobenzidine (DAB), which produced a brown color. Haematoxylin was used for counterstaining. The localization of P53-expressing cells was evaluated in the nucleus and/or cytoplasm, and sections were classified as positive or negative.

### Statistical Analysis

Data are represented as mean ± SD. A one-way ANOVA (Analysis of Variance) was employed to compare the mean values of various variables among different groups. When the ANOVA showed a significant difference, post hoc tests (Tukey’s Honest Significant Difference, HSD) were used to determine which specific groups differed from each other. A significant p-value (*p* < 0.05) indicates significant differences between the group means. All data were analyzed using Minitab version 21 (LLC), GraphPad Prism version 8.3, and Microsoft Excel 365 (Microsoft Corporation, USA).

The mean area percentage of P53 immunoexpression was measured using an image analysis system (Leica DM LB2 with QWIN plus image analyzer computer system, Germany). The obtained data were calculated, analyzed, and compared using the ANOVA test to assess changes in the area percentage of P53 immunoexpression between the studied groups. Results were considered significant at *p* ≤ 0.05 and highly significant at *p* ≤ 0.01.

## Results

### In Vitro Assay

For the accurate assessment of the in vitro effect of the LC90 of *C. andromeda* venom, adult worms and larvae were collected from the intestines and skeletal muscles, respectively, of infected mice immediately after dissection The cultivated adults and larvae of *T. spiralis* were examined by mechanical shock with a needle to assess mortality. They were completely dead after ten hours of exposure to the venom.

### Scanning Electron Microscopy of Adults and Larvae of *T. spiralis* (Figs. 2 and 3)

**Fig. 2:**
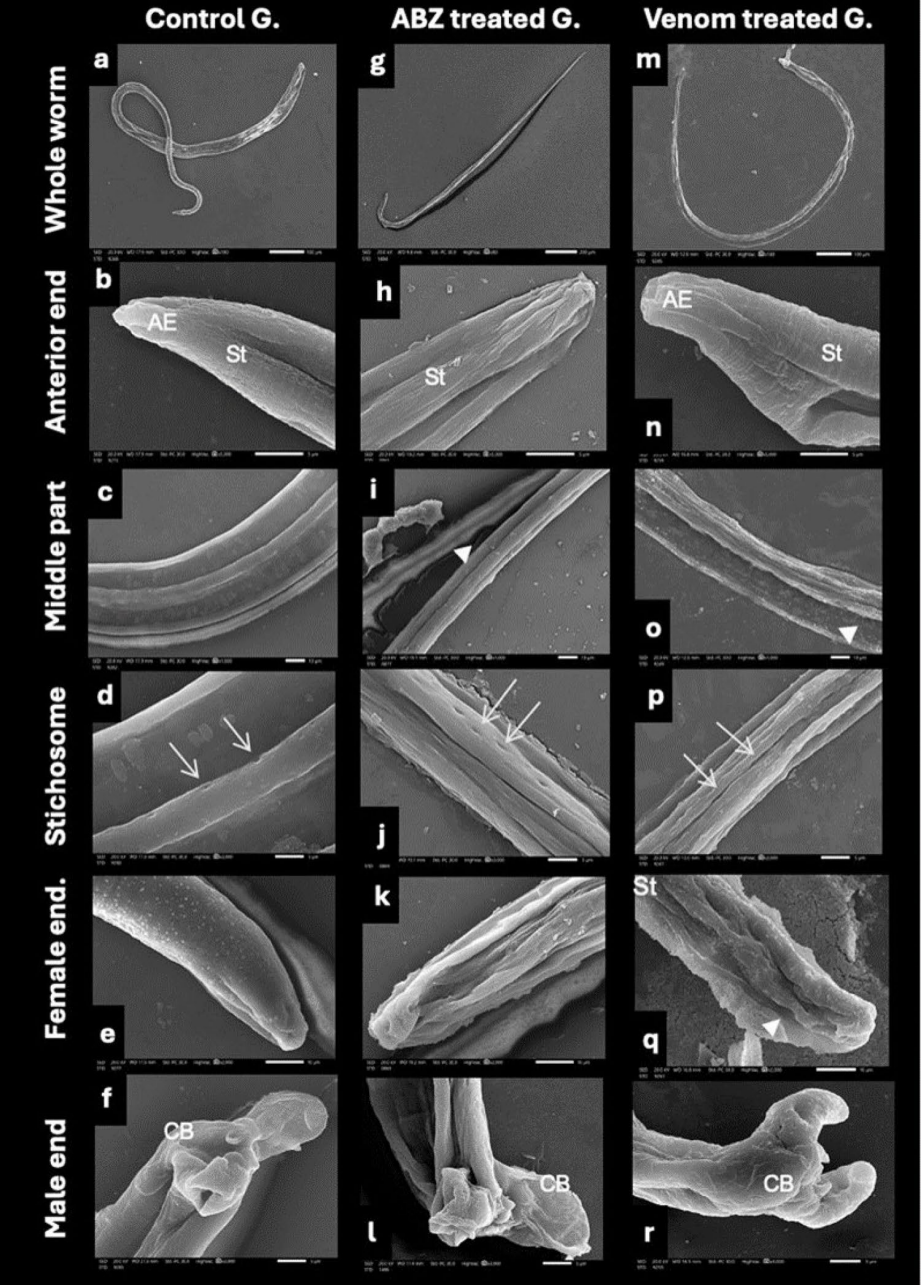
Scanning electron micrographs reveal the ultrastructural alterations in the adult *T. spiralis* during the acute phase. **a**–**f** Control adult worm shows smooth striated cuticle (St), pointed anterior end (AE), systematic arrangement of stichosomes (arrows), and an intact appearance of female ends (s) and male’s copulatory bursae (CB). **g**–**l** Adults treated with albendazole show noticeable shrinkage and loss of striations, prominent deep longitudinal creases (arrowheads), irregular stichosome arrangement (arrows), and significant shrinking of the female end and male copulatory bursa. **m**–**r** Adults treated with venom exhibit noticeable swelling, deep longitudinal creases (arrowheads), flattening of the anterior end and loss of striations. Note the embedding of stichosomes between the longitudinal creases of the cuticle (arrows), the swelling and corrugation of the female end and cracking of the male’s copulatory bursa

**Fig. 3:**
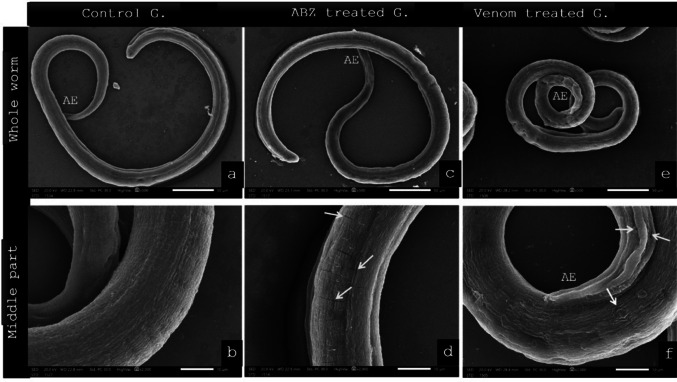
Scanning electron microscopy reveals the ultrastructural alterations in *Trichinella spiralis* larvae during the chronic phase. **a**, **b** Untreated control larvae (G II) show a smooth somatic cuticle. **c**, **d** Larvae treated with albendazole (G IV) exhibit minor longitudinal creases. The cuticle displays transverse and longitudinal cracks (red arrow) and is shrunken. **e**, **f** Larvae treated with jellyfish venom exhibit significant swelling, characterized by a bumpy, bubbling (yellow arrow) and corrugated surface with ruptures (blue arrow). The anterior end (AE) has deep longitudinal creases or cuticle folds with ruptures

Scanning electron microscopy was used to examine ultrastructural alterations in *T. spiralis* adults following in vitro exposure to *C. andromeda* venom .The investigation compared specimens exposed to the LC90 of *C. andromeda* venom with those cultivated in albendazole (reference drug) and RPMI medium only (control group).

Examination of the control group revealed adult *T. spiralis* with a normal size (Female; 3.5 × 0.76 mm, male;1.5 × 0.05 mm) [53], a smooth and well-defined striated cuticle, systematically arranged stichosomes, and a structurally intact copulatory bursa in males. On the other hand, albendazole-treated worms exhibited detectable shrinkage, cuticular striation distortions, and deep longitudinal creases. Stichosomes appeared disfigured; both the female posterior end and the male copulatory bursa showed marked shrinkage and deformation. Venom-treated adults showed body swelling, disrupted cuticular striations with pronounced longitudinal creases, and a flattened anterior end. Furthermore, stichosomes appeared blurred and embedded within the creases, the female posterior end showed swelling and corrugation, and the male copulatory bursa exhibited signs of inflammation and structural disruption.

Examination of the *T. spiralis* larvae during the chronic phase showed a smooth, normal, striated cuticle. Larvae treated with albendazole exhibited minor longitudinal creases throughout their bodies. The cuticle had shrunken and displayed transverse and longitudinal cracks. Larvae treated with jellyfish venom exhibited significant swelling, characterized by a bumpy and corrugated surface with ruptures. The anterior end exhibited deep longitudinal creases and ruptures.

### In Vivo Assay

#### Body Weight

**Fig. 4:**
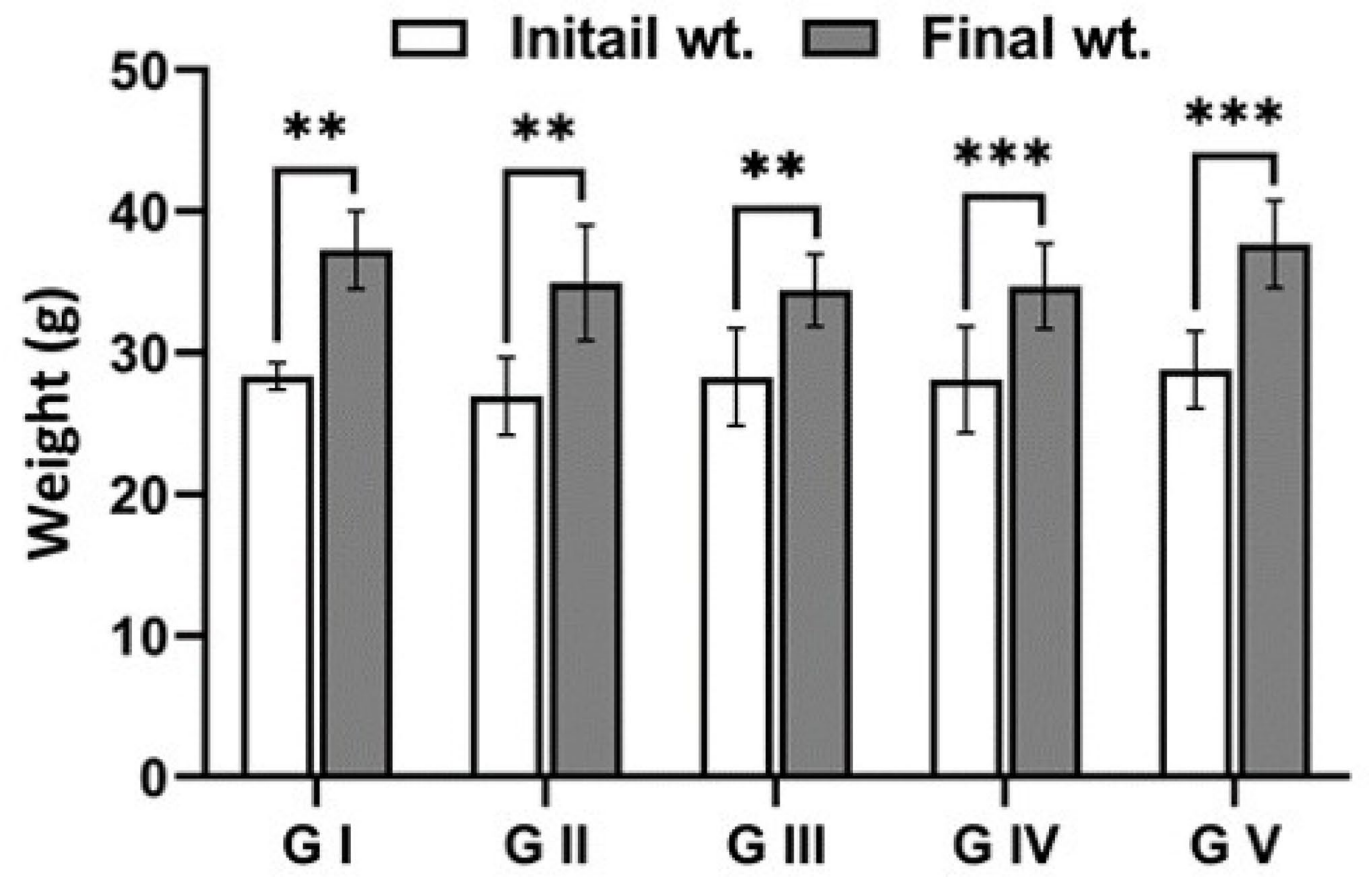
Body weight of mice for the experimental groups of the intestinal phase: G (I) uninfected untreated group (control group), G (II) infected with *T. spiralis* and untreated, G (III) uninfected and treated with jellyfish venom, G (IV) infected with *T. spiralis* and treated with albendazole and G (V) infected with *T. spiralis* and treated with venom. The body weight was measured at the beginning of the experiment (initial weight) and at the 7th dpi (final weight). ** *p* < 0.01, *** *p* < 0.001

**Fig. 5:**
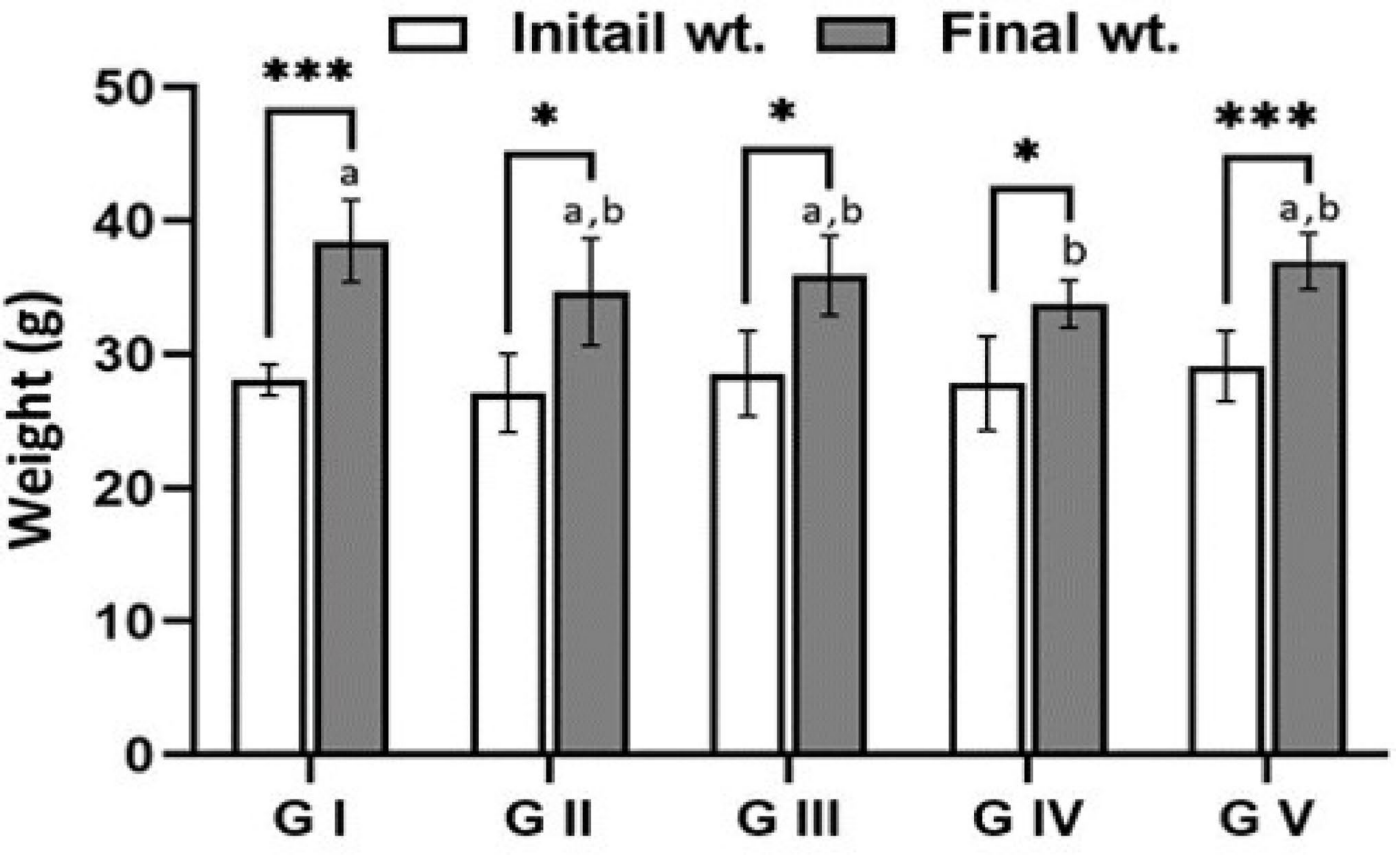
Body weight of mice for all groups of the chronic phase: G (I) uninfected untreated group (control group), G (II) infected with *T. spiralis* and untreated, G (III) uninfected and treated with jellyfish venom, G (IV) infected with *T. spiralis* and treated with albendazole and G (V) infected with *T. spiralis* and treated with venom. The body weight was measured at the beginning of the experiment (initial weight) and at the 36th dpi (final weight). * *p* < 0.05, *** *p* < 0.001

The investigation of all groups in both the acute and chronic phases showed that the mice had almost identical activity and feeding patterns. Figures 4 and 5 indicate that there were no significant differences in body weight between the groups; the control group gained the most weight, followed by the infected group treated with venom (G V), while the infected group (G III) showed the least weight gain.

#### Parasitological Assessment

The present results demonstrate statistically significant differences (*p* < 0.001) in the mean number of *T. spiralis* adults and larvae among the three infected groups (G II, G IV, G V), indicating that the treatments considerably reduced the parasite burden compared to the control. The venom-treated group exhibited a significantly lower recovery rate for both stages, with reductions of 85.23% and 95% for adults and larvae, respectively. These values were markedly lower than those observed in the albendazole-treated group (Table 1).

**Table 1:** Counts of *T. spiralis* adults and larvae in the infected groups during the acute and chronic phases

Group	Acute phase		Chronic phase	
	Adult count	Reduction %	Lava count	Reduction %
G II	71.8 ± 4.21^a^	–	13,363 ± 1677^a^	–
G IV	15.6 ± 2.3^b^	78.27%	3840 ± 887^b^	63.20%
G V	10.6 ± 3.58^b^	85.23%	522.2 ± 121.4^c^	95%
*p*-value	< 0.001	–	< 0.001	–

Groups with different letters are significantly different from each other (*p* < 0.05)

#### Haematological Investigation

The complete blood count (CBC) for all groups in both the acute and chronic phases are illustrated in Tables 2 and 3. In the infected untreated group (G II), the total counts of RBCs and PLTs showed significant reductions (*p* < 0.05) compared to those in the control group. In contrast, the treated groups (G IV, G V) exhibited a significant increase (*p* < 0.05) compared to the infected group. Meanwhile, the levels of Hb% and HCT% decreased with no significant difference among the different groups. The total count of WBCs increased significantly (*p* < 0.05) in group G II compared to the control group (G I), whereas the treated groups showed a non-significant decrease relative to the infected group (G II).

**Table 2:** Complete blood count (CBC) among different experimental groups in the acute phase at 7th dpi

Acute phase	RBCs	WBCs	Hb%	HCT%	PLT
G I	8.06 ± 1^a^	3.23 ± 0.68^b^	13.56 ± 3.46	34.73 ± 2.51	1260 ± 191^a^
G II	4.3 ± 0.76^b^	10.64 ± 3.83^a^	8.13 ± 2.3	23.63 ± 3.35	248 ± 44.9^c^
G III	7.63 ± 0.81^a^	5.8 ± 3.57^a, b^	12.47 ± 2.4	28.97 ± 7.87	934 ± 32.1^b^
G IV	7.65 ± 0.65^a^	8.83 ± 1.63^a, b^	11.43 ± 1.45	33.33 ± 6.82	835 ± 30.4^b^
G V	7.19 ± 0.92^a^	6.75 ± 0.09^a, b^	11.5 ± 1.61	31.87 ± 5.2	765.3 ± 127.7^b^
*p-*value	0.002	0.035	0.139	0.188	< 0.001

Groups with different letters are significantly different from each other (*p* < 0.05)

**Table 3:** Complete blood count (CBC) among different experimental groups in the chronic phase at 36th dpi

Chronic phase	RBCs	WBCs	Hb%	HCT%	PLT
G I	10 ± 0.37^a^	4.28 ± 0.53^b^	14.55 ± 0.27^a^	41.95 ± 2.68	1049.5 ± 129.4^a^
G II	7.27 ± 0.48^c^	7.96 ± 1.95^a^	11.33 ± 1.13^c^	36.55 ± 5.26	711.3 ± 161.1^b^
G III	9.72 ± 0.55^a^	4.47 ± 1.72^b^	14.15 ± 0.55^a, b^	38.53 ± 1.87	946.8 ± 41.2^a, b^
G IV	8.51 ± 0.49^b^	4.99 ± 1.71^a, b^	11.98 ± 0.99^b, c^	40.35 ± 1.81	913.5 ± 58.2^a, b^
G V	8.62 ± 0.39^b^	4.78 ± 0.83^a, b^	12.53 ± 1.93^a, b,c^	39.98 ± 6.34	890.3 ± 125.6^a, b^
*p*-value	< 0.001	0.016	0.004	0.431 n.s.	0.011

Groups with different letters are significantly different from each other (*p* < 0.05)

#### Biochemical Analysis

##### Liver Enzymes and Total Protein Levels

**Fig. 6:**
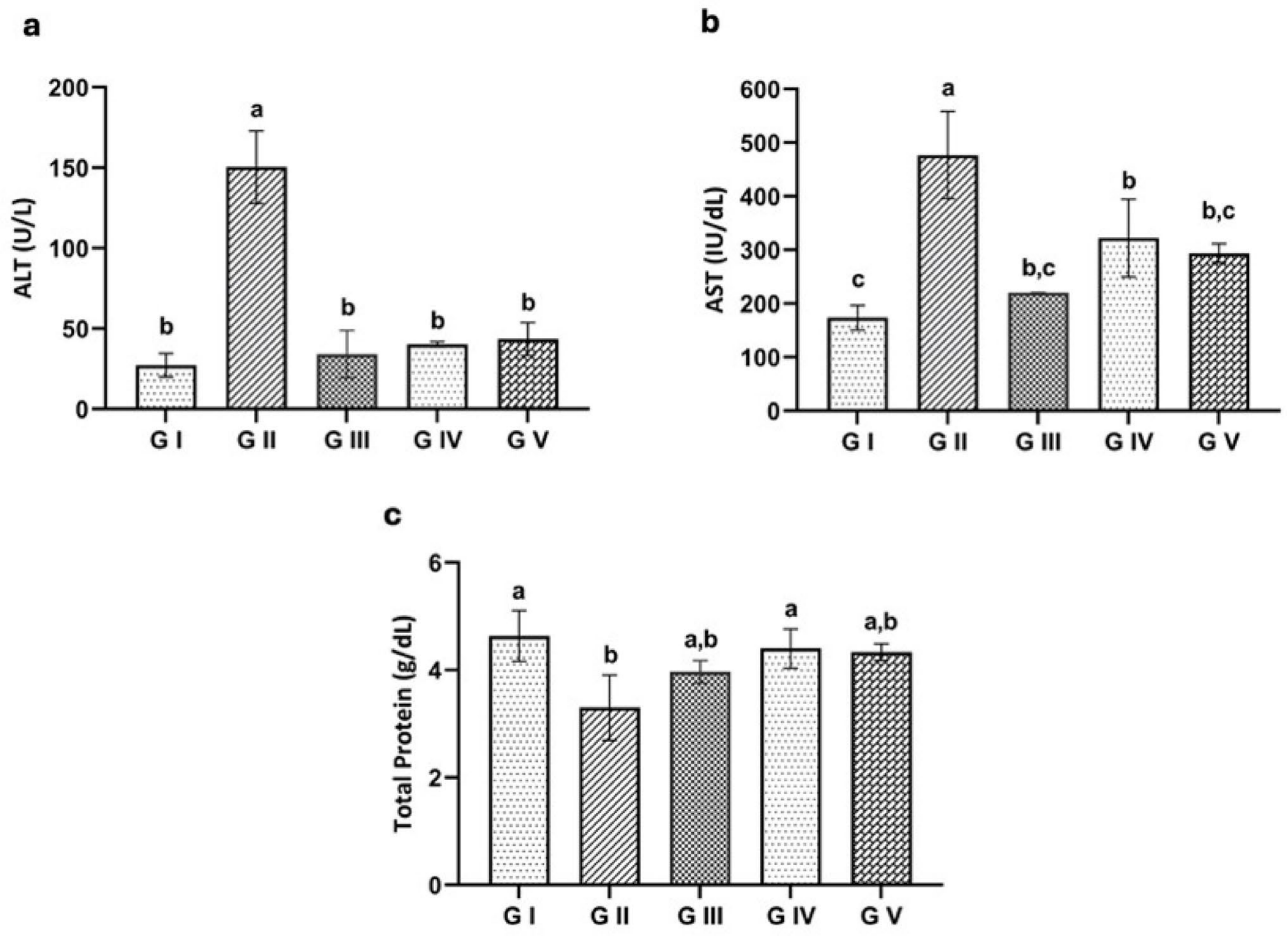
Liver enzyme levels in the experimental groups at the acute phase. **a** Alanine aminotransferase (ALT), **b** Aspartate aminotransferase (AST), **c** Total protein. Groups with different letters are significantly different from each other (*p* < 0.05)

**Fig. 7:**
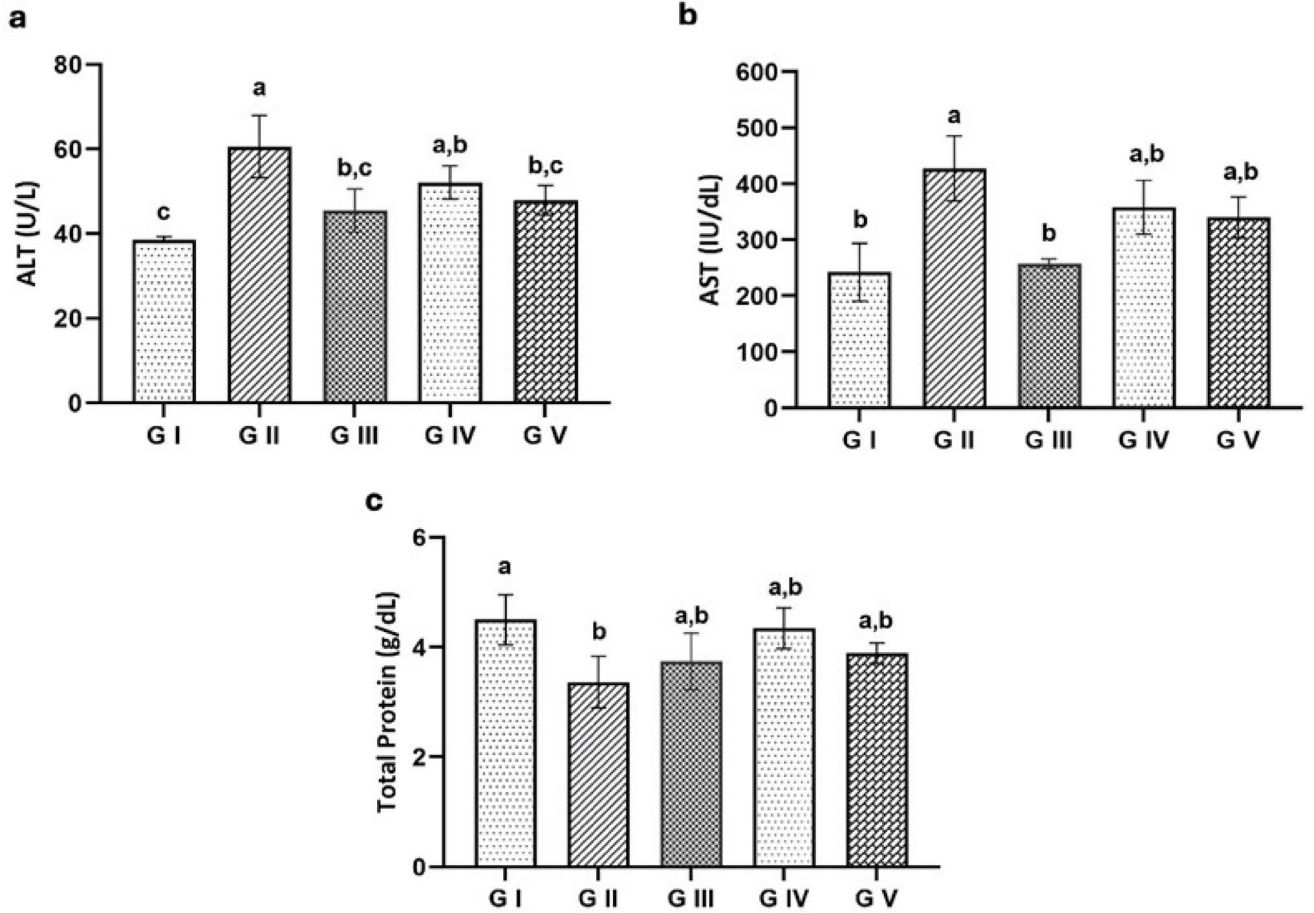
Liver enzyme levels in the experimental groups at the chronic phase. **a** ALT, **b** AST, **c** Total protein. Groups with different letters are significantly different from each other (*p* < 0.05)

Infection with *T. spiralis* caused a significant increase in serum alanine aminotransferase (ALT) and aspartate aminotransferase (AST) levels during the acute and chronic phases compared to the uninfected control group (*P* < 0.001). In contrast, the treated groups showed a notable decrease in these enzyme levels when compared to the infected, untreated group (*P* < 0.001) (Figs. 6 and 7a, amp and b). The results in Figs. 6c and 7c revealed a significant decrease (*P* < 0.05) in total protein levels in the infected untreated group (G II) compared to the uninfected control, with a detectable increase in total protein in the two treated groups (G IV and G V) relative to the infected untreated group.

##### Oxidative Stress Biomarkers

**Fig. 8:**
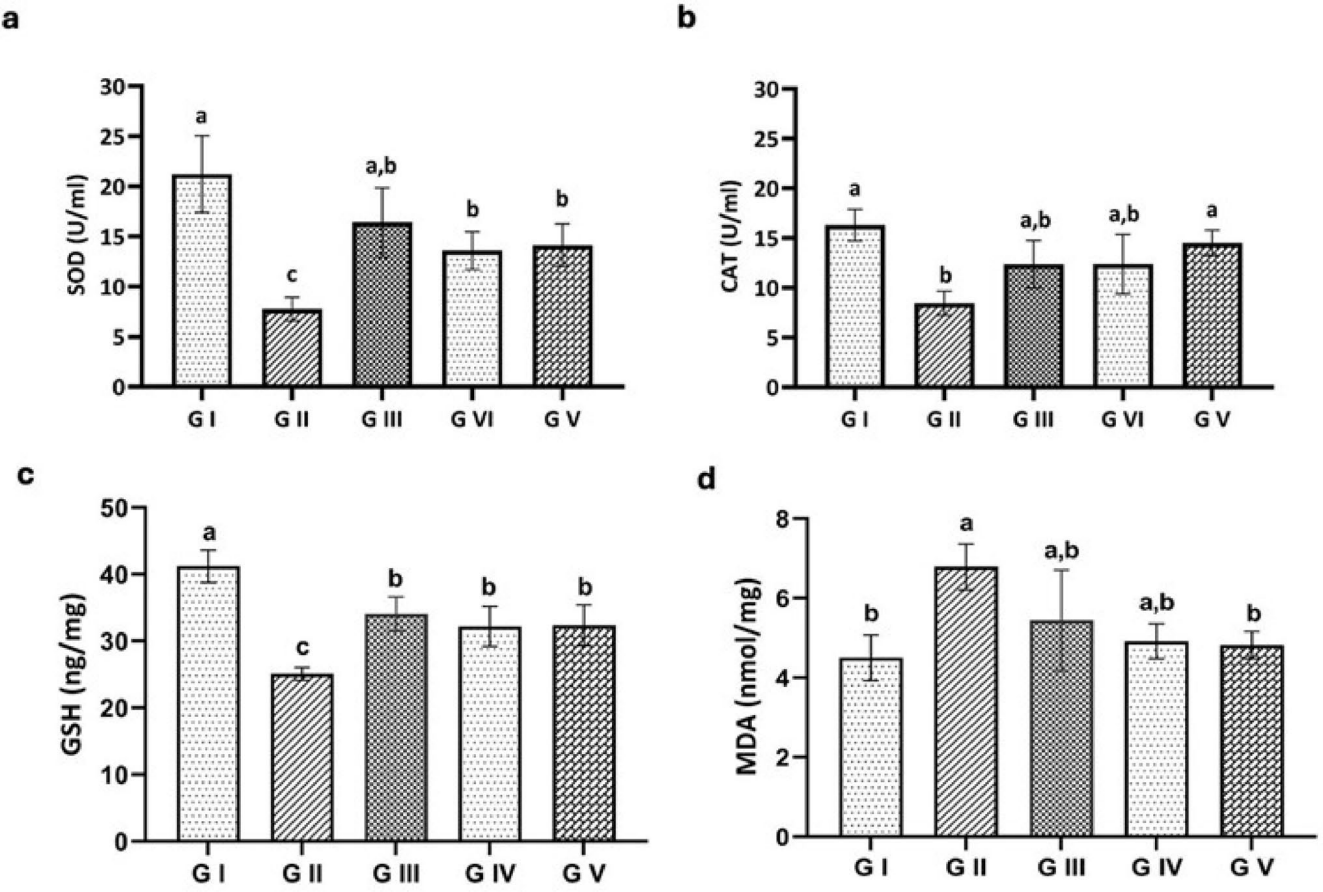
Oxidative stress biomarker levels in the experimental groups at 7th dpi. **a** Catalase (CAT), **b** Superoxide dismutase (SOD), **c** Glutathione peroxidase (GSH-Px), **d** Malondialdehyde (MDA). Groups with different letters are significantly different from each other (*p* < 0.05)

**Fig. 9:**
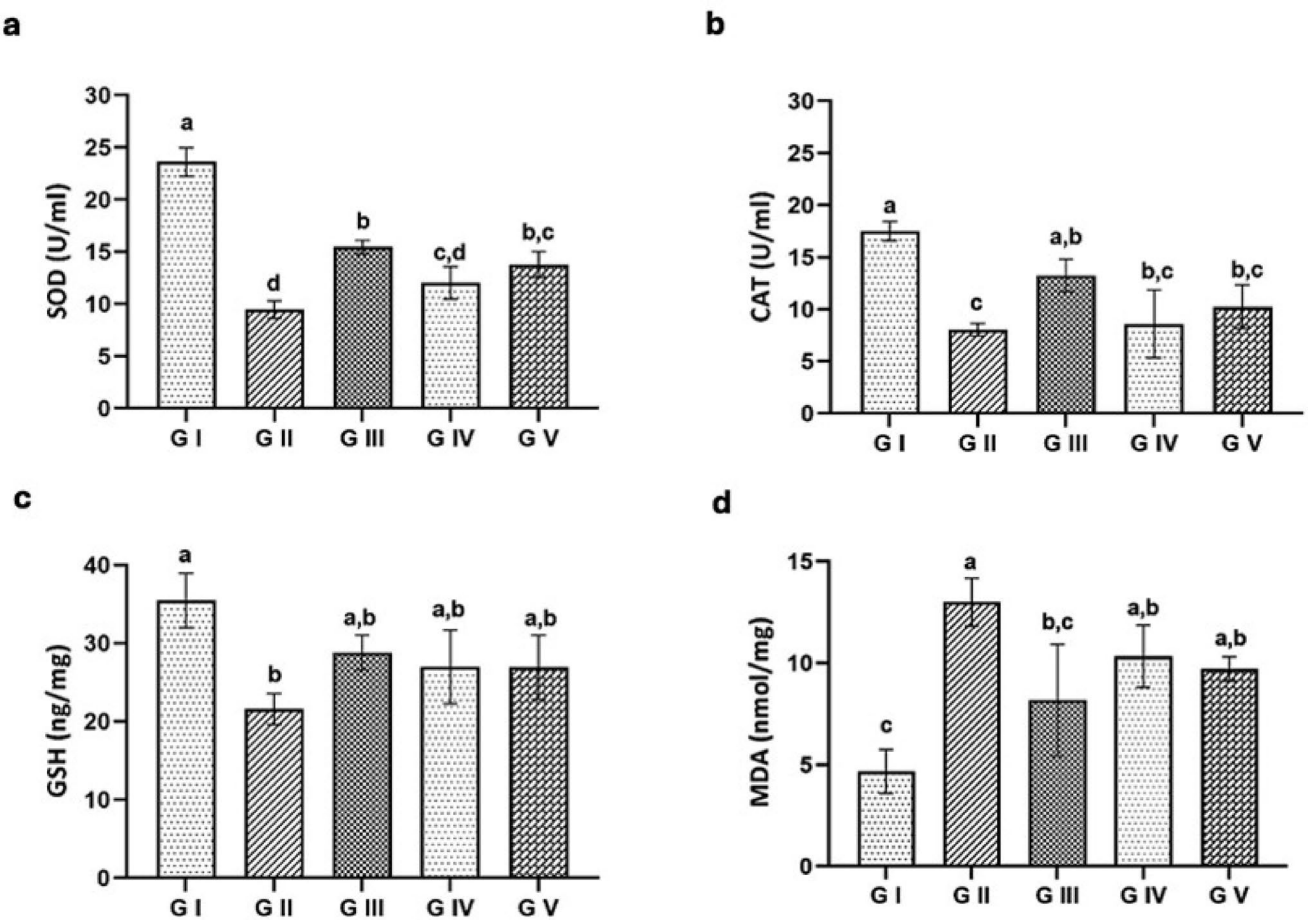
Oxidative stress biomarker levels in the experimental groups at 36 dpi. **a** CAT, **b** SOD, **c** GSH-Px, **d** MDA. Groups with different letters are significantly different from each other (*p* < 0.05)

In both the acute and chronic phases, the infected untreated group (G II) exhibited a marked decrease (*p* < 0.001) in SOD, CAT, and GSH activities, alongside a significant increase (*p* < 0.001) in MDA levels when compared to the control group (G I). The albendazole-treated group (G IV) showed a significant rise (*p* < 0.001) in GSH, with SOD and CAT activities remaining non-significantly increased and a non-significant drop in MDA levels compared to the infected untreated group. Furthermore, the infected group treated with venom (G V) showed a significant increase (*p* < 0.05) in SOD, CAT, and GSH activities and a significant decrease (*p* < 0.001) in MDA levels compared to the infected untreated group (Figs. 8 and 9).

##### Interleukin-6 Level Among Different Groups of Mice

**Fig. 10:**
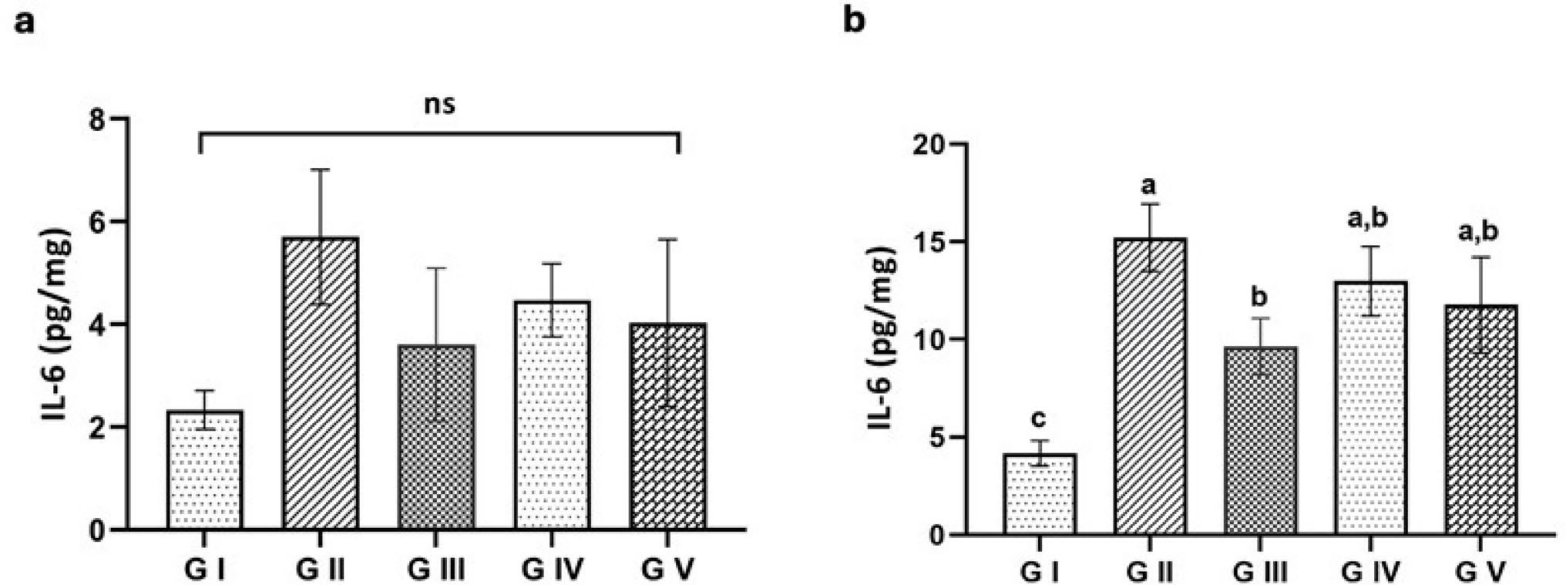
Interleukin-6 (IL-6) level among experimental groups in: **a** the intestinal tissues at 7th dpi, **b** the skeletal muscles at 36th dpi. ns. not significant

Figure 10a shows the IL-6 levels in the small intestine tissue of different experimental groups. The highest level (5.7 ± 1.31) was observed in the infected untreated group (G II) compared with the control group (G I) level (2.33 ± 0.38). Treated groups G III, G IV, and G V showed an insignificant decrease in IL-6 levels relative to the infected untreated group (G II). IL-6 levels in the skeletal muscle tissue were significantly increased in the infected group (G II). Nevertheless, they were significantly decreased in the treated groups G IV and G V compared with the infected group and demonstrated no significant variation compared to the control group (Fig. 10b).

#### Histopathological Alterations in Intestinal and Skeletal Muscle Tissues

##### Small Intestine

**Fig. 11:**
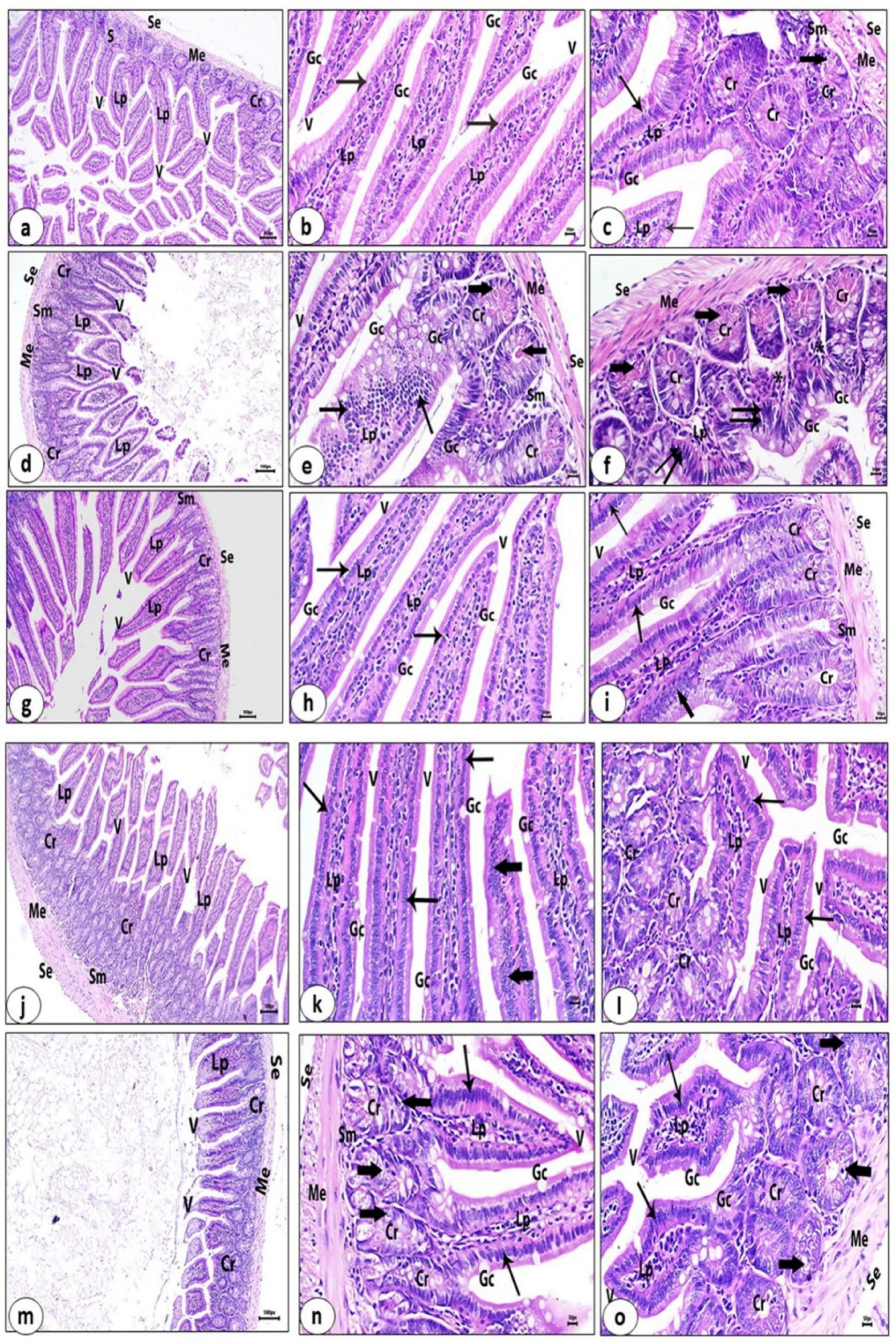
Histological sections of the small intestine of different experimental animal groups stained with haematoxylin and eosin. Uninfected control group (G I): **a** serosa (Se), muscularis externa (Me), submucosa (Sm), cross sections of crypts (Cr) and villi (V). (X 100). **b** and **c** Tall regular villi (V), columnar lining epithelium with a straight border (arrows), goblet cells (Gc), Lamina propria (Lp), and Paneth cells (thick arrow) (X 400). Infected untreated group (G II): **d**, **e** and **f** Disorganized villi (V) (arrows), hyperplasia of goblet cells (Gc), shortened villi (double arrows), dysplasia of mucosal glands (Cr), accumulation of Paneth cells secretions (thick arrows) and dense inflammatory cellular infiltrate (_*_) (X 100, 400). Uninfected and venom-treated group (G III): **g**, **h** and **i** Normal villi (V); normal enterocytes (arrows), regular goblet cells (Gc), hyperplasia (thick arrow), and organized crypts (Cr) (X 100, 400). Infected albendazole-treated group (G IV): **j**, **k** and **l** Enhanced architecture of villi (V), intact lining epithelium (arrows, hyperplasia (thick arrows) and proliferated crypts (Cr) (X 100, 400). Infected venom-treated group (G V): **m**, **n** and **o** Regular tall villi (V), normal lining epithelium (arrows) and normal goblet cells (Gc), crypts (Cr) with a limited number of vacuolated epithelial cells (thick arrows) (X 100, 400)

Examination of the control group’s (G I) small intestine displays normal features, including the serosa layer, the muscularis externa, submucosa and mucosa layer that contains tall, regularly arranged villi, cross sections of the intestinal glands (crypts), and strips of smooth muscle fibers which are extended between the intestinal glands (Fig. 11a). The previously mentioned section’s high magnification revealed tall, regular villi with columnar lining epithelium with straight borders and goblet cells, as well as a core of lamina propria that contained small blood vessels and widespread lymphatic tissue. Crypts exhibit Paneth cells with brightly eosinophilic cytoplasmic globules (Figs. 11b, c).

Sections of the small intestine from the infected untreated control group (G II) displayed significant disarray, especially in the mucosa and submucosa layers. These were characterized by disorganized villi with hyperplasia of its lining epithelium and increased number of goblet cells, others are atrophied, shortened with loss of their height, dysplasia of mucosal glands (crypts) with hyperchromatic epithelial cells (pyknotic nuclei), accumulation of Paneth cell secretions, and dense inflammatory cellular infiltration in the lamina propria (Figs. 11d–f). Conversely, the uninfected and venom-treated group (G III)’s small intestine sections show a normal-looking structure, which is characterized by normal tall villi, normal lining enterocytes, and normal regular goblet cells. A few number of the villi exhibit hyperplasia of their lining epithelia, and mucosal glands are organized; these were also seen (Figs. 11g–i).

Figure 11j–l show low- and high-magnified sections of the small intestine of the infected group treated with albendazole (G IV) that exhibited improvement in its structure, which is defined by the presence of finger-like villi with apparently intact lining epithelia. A few of them have hyperplasia of their lining epithelia. Furthermore, the proliferation and hyperplasia of crypts were noticed.

Examination of the small intestine of the infected and venom-treated group (G V) exhibited pronounced improvement of the small intestine characteristics, which are proven by villi of regular length with normal lining epithelia, normal and regular goblet cells, and organized mucosal glands (crypts) that revealed few histopathological changes like limited numbers of their lining epithelia with vacuolated or degenerated cytoplasm (Figs. 11m–o).

##### Skeletal Muscles

**Fig. 12:**
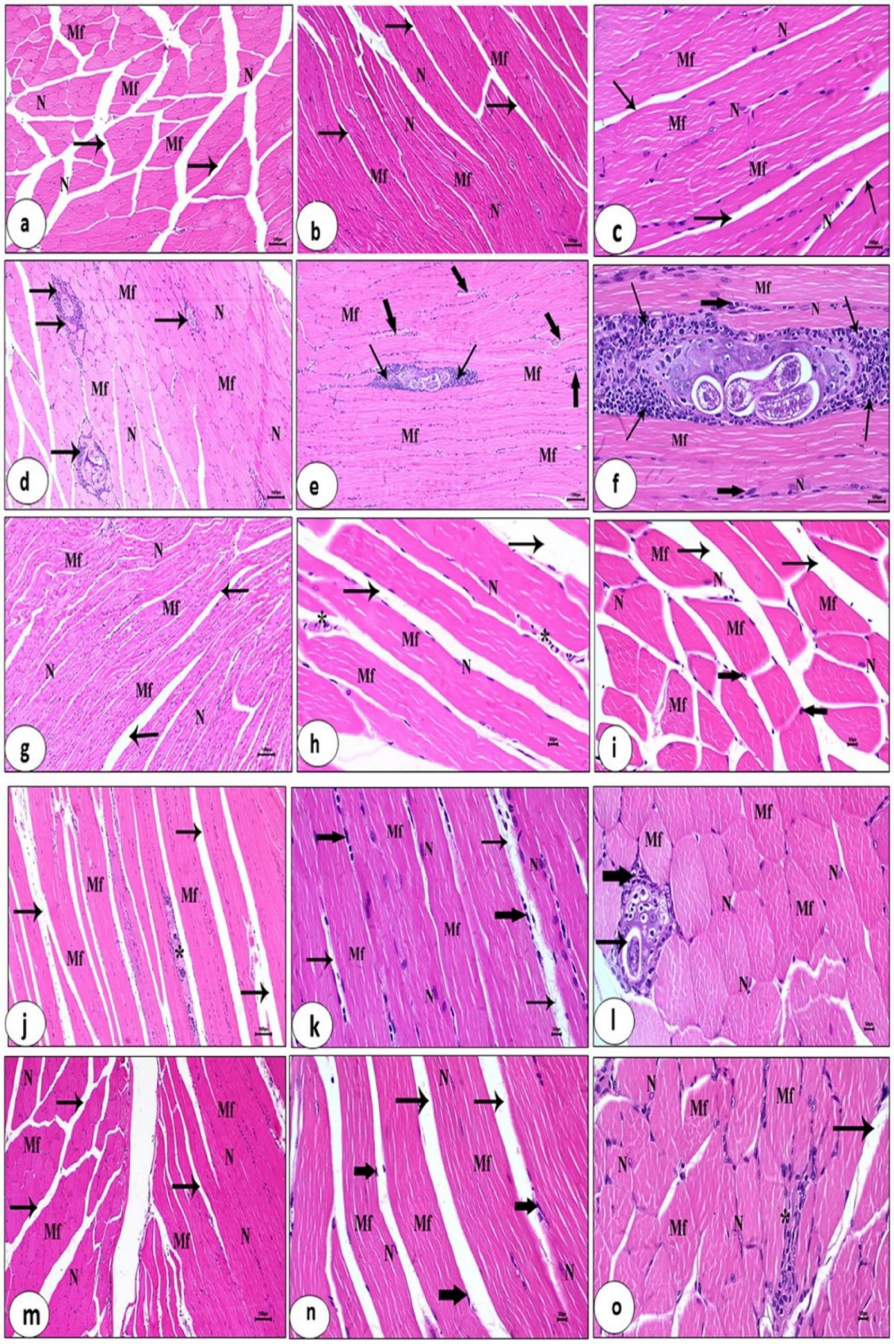
L.S. and T.S. of skeletal muscles of different experimental animal groups stained with haematoxylin and eosin. Uninfected control group (G I): **a**, **b** and **c** Parallel striated muscle fibers (myocytes) (Mf) with peripherally intact nuclei (N) and regular endomysia (arrows) (X 100,100,400). Infected untreated group (G II): **d**, **e** and **f** Disarrangement of myocytes (Mf), numerous larvae within intact capsules surrounded by dense inflammation (arrows), and endomysium with infiltrated leukocytes (thick arrows) (X 100,100,400). Uninfected and venom-treated group (G III): **g**, **h** and **i** Myocytes (arrows), leukocytic infiltration (*) (X 100,400,400). Infected albendazole-treated group (G IV): **j**, **k**, **l** Necrosis of larvae, disruption of the capsule, and penetration of inflammatory cells (_*_), and dilated myocytes (arrows) with minimal inflammatory cells (thick arrows) (X100,400,400). Infected venom-treated group (G V): **m**, **n** and **o** Enhanced myocytes (Mf), degenerated larvae with incomplete capsule, invaded by few inflammatory cells (_*_), myocytes with pyknotic nuclei (thick arrows) and dilatations of endomysia (arrows) (X 100,400)

Light microscopic examination of skeletal muscles of the control uninfected group (G I) displayed well-organized parallel striated muscle fibers (myocytes) with an evident transversely striated pattern, peripherally located normal intact nuclei and regular endomysia between myocytes (Figs. 12a-c). The infected untreated group (G II) showed disorganization of the muscular tissue, disarrangement of the myocytes, numerous larvae embedded in the muscles within intact capsules that are surrounded by dense inflammation composed of lymphocytes, plasma cells and few macrophages as well as deteriorated endomysia with infiltrated leukocytes (Figs. 12d-f). Figures 12g-i showed sections of skeletal muscles of the uninfected and venom-treated group (G III) that exhibited normal-like architecture of the muscular tissue, normal appearance of the myocytes with normal oval nuclei approximately similar to normal control status, except for distancing of myocytes and minimal leukocytic infiltration in the endomysia was noticed. Sections of skeletal muscles of the infected group treated with albendazole (G IV) exhibited improvement of the muscular tissue that was represented by necrosis of larvae, disruption of the capsule, and penetration of inflammatory cells within the capsule, but some histopathological changes were observed such as dilatation and distancing of the myocytes with minimal inflammatory cells (Figs. 12j -l). Sections of skeletal muscles of infected and venom-treated group (G V) exhibited obvious improvement and restoration of the histological structure with normal appearance of the myocytes, degeneration and necrosis of the remaining larvae with broken down incomplete capsules, which are completely invaded and surrounded by few inflammatory cells. A few no of myocytes with dense chromatin nuclei (pyknotic nuclei) and dilatation of endomysia were observed (Figs. 12m-o). Treated mice muscles showed a decrease in larval count and amelioration in the inflammatory tissue.

#### Immunohistochemical Study

##### Small Intestine

**Fig. 13:**
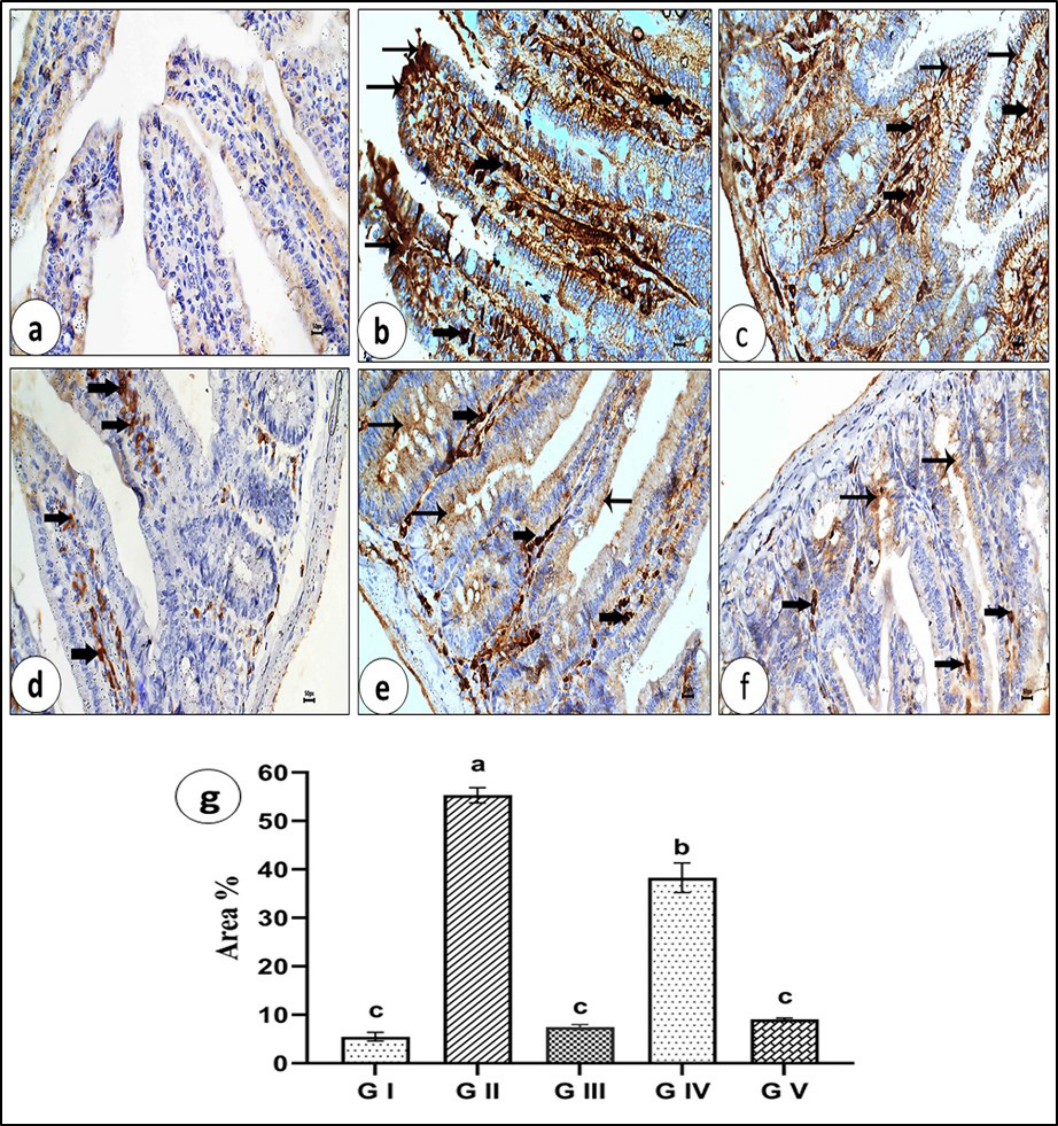
P53 immunostaining in the small intestine sections of different experimental groups (X400): **a** Section of uninfected control group (G I) آegative immunoreactivity to P53 immunostain in the enterocytes and inflammatory cells in the lamina propria and submucosa. **b** and **c** Sections of infected untreated group (G II) Strong positive reaction to P53 immunostain in the cytoplasm of enterocytes (arrows) and macrophages or stromal cells (thick arrows). **d** Section of uninfected and venom-treated group (G III) Absence of P53 immunostaining except a weak stain localized in the macrophages in the lamina propria (thick arrows). **e** Section of infected and albendazole treated group (G IV) Few numbers of enterocytes (arrows) and macrophages (thick arrows) are still seen with weak immunostaining to P53. **f** Section of infected and venom-treated group (G V) Noticeable improvement without immunostaining of P53, except for a few enterocytes (arrows) and macrophages (thick arrows). **g** Mean area percent of P53 immunoexpression

The immunohistochemical expression of the apoptotic marker P53 among the five experimental groups reveals distinct patterns. The expression of p53 and the incidence of apoptotic cells were very low or nearly absent in the small intestine sections of the control group (G I) (Fig. 13a). In contrast, G II exhibited strong P53 immunohistochemical expression and high cytoplasmic staining intensity in the enterocytes of the small intestine sections and macrophages in the lamina propria (Figs. 13b, c). The small intestine sections of G III revealed negative immunoreactivity to P53 in the enterocytes, with low or mild immunoreactivity in the macrophages or stromal cells of the lamina propria (Fig. 13d). Conversely, G IV illustrated moderate immunohistochemical expression in both the enterocytes and the macrophages (Fig. 13e). G V showed a significant improvement, marked by a reduction in P53 immunostaining in the small intestine sections, although a few enterocytes and macrophages exhibited low P53 immunoexpression (Fig. 13f).

The lowest mean area percent of P53 immunoexpression was observed in G III and G V, with no significant difference between them and the control group (G I), whereas the highest value was recorded in G II. The analysis of variance (ANOVA) test revealed that the difference between the studied groups was highly significant (*p* ≤ 0.001) (Fig. 13g).

##### Skeletal Muscle

**Fig. 14:**
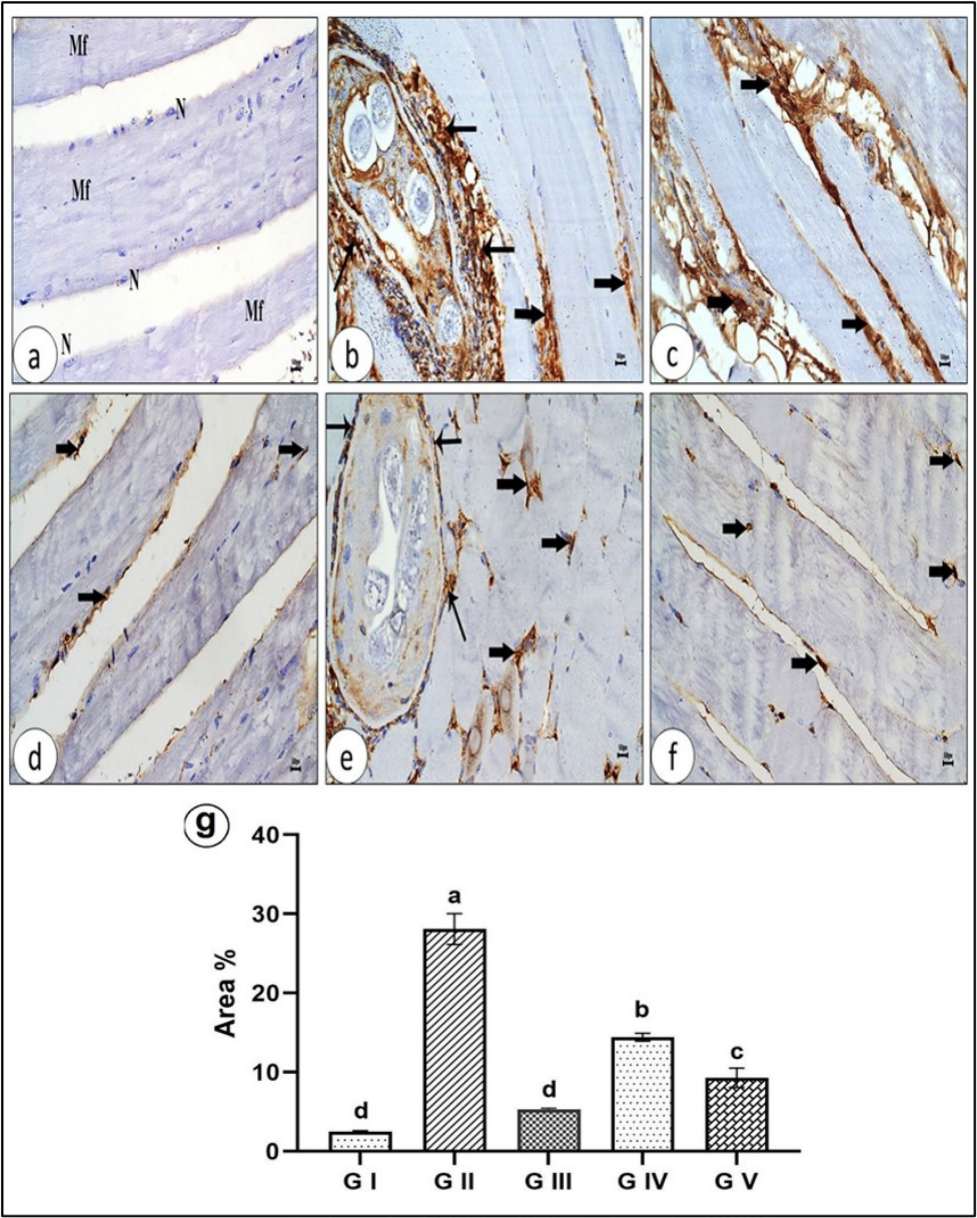
P53 immunostaining in the L.S. of skeletal muscle of different experimental groups (X400): **a** Uninfected control group (G I) Approximately absence of immunostaining to P53 in the myocytes (Mf). **b** and **c** Infected untreated group (G II) Marked intense immunostaining to P53 in the dead myocytes and interstitial spaces between muscle bundles (arrows) and cellular infiltrates surrounding larva (thick arrows). **d** Uninfected and venom-treated group (G III) Absence of immunostaining to P53, except a faint stain in the nuclei of myocytes (thick arrows). **e** Infected and albendazole-treated group (G IV) Few myocytes (thick arrows) and inflammatory cells (thin arrows) are still seen with P53 immunostaining. **f** Infected and venom-treated group (G V) Disappearance of P53 immunostaining in the myocytes, except weak nuclear immunostaining in few cells (thick arrows). **g** Mean area percent of P53 immunoexpression**.**

In the chronic phase, the expression of P53 in the myocytes of control mice expressed a very weak immunostaining localized in the sarcoplasm and the nuclei of myocytes (Fig. 14a). Positive untreated group (G II) showed a marked intense of P53 immunostain was expressed in the sarcoplasm of myocytes, interstitial spaces between muscle fibers and cellular infiltrates surrounding larva (Figs. 14b, c). Figure 13d illustrated absence or approximately disappearance of P53 in the myocytes of skeletal muscles of non-infected and venom-treated group (G III) nearly similar to normal control mice group, except few numbers of myocytes exhibited weak immunoreactivity to P53 immunostaining. While skeletal muscles of infected group treated with albendazole (G IV) illustrated partial improvement, few numbers of myocytes and inflammatory cells surrounding degenerated larva with absent of capsule are still seen with moderate P53 immunostain reactivity (Fig. 14e). Figure 14f revealed noticed improvement and obviously disappearance of P53 immunostain expression in the myocytes of skeletal muscles of infected venom-treated group (G V).

## Discussion

Humans are among the variety of mammalian hosts infected by the global nematode *T. spiralis*, which can cause potentially fatal consequences [1]. Due to the limitations of benzimidazole derivatives in treating the parasite’s encapsulated larval stages, medical researchers are actively searching for new, safe, and effective anthelminthic agents.

Marine organisms’ venoms are being developed as potential sources of unique antimicrobials which have potent antimicrobial properties. Cnidarians, such as jellyfish, produce venom through specialized nematocytes, which contain protein-based toxins with immunomodulatory and physiological effects [54, 55]. Studies on jellyfish venoms as nematocidal agents are still in their initial phases, with previous investigations exploring the in vitro effects of *C. andromeda*’s venom on *Toxocara canis* third-stage larvae [33] and of *Rhopilema nomadica*’s venom on adult *T. spiralis* [56].

Albendazole is a benzimidazole anthelmintic that is often administered orally due to its poor water solubility and requirement for gastrointestinal absorption [57]. However, the venom was injected to prevent gastrointestinal breakdown and ensure systemic exposure to its bioactive constituents, because many cnidarian venom proteins and enzymes are highly susceptible to proteolytic inactivation when taken orally [58, 59]. The goal of the current study is to compare the biological effects of two different treatment approaches: a new injectable marine bioactive extract and a traditional oral chemotherapeutic drug on adult and larval stages of *T. spiralis* during the acute phase of a murine infection. Although variations in dosage and administration method create inherent limitations to direct comparisons, they nevertheless offer important initial information about the potential of C. andromeda venom as a nematocidal agent.

The parasitological assessment revealed that the highest adult worm count of *T. spiralis* was detected in the infected untreated group of mice, comparable to findings of Salama et al. [60] and El-Saidy et al. [61]. On the other hand, treated groups of mice exhibited a significantly lower recovery rate of adult worms, with reduction percentages of 78.27% in the infected and ABZ-treated group and 85.23% in the infected and venom-treated group. In fact, the anthelminthic impact of *C. andromeda*’s venom could be related to the complexity and significance of its marine toxin contents. Neurotoxic peptides, bioactive lipids, and proteinaceous porins in venoms can create pores in the cellular membranes [62–64]. Furthermore, saturated fatty acids, particularly palmitic, decanoic and nonanoic acids, are well known to exhibit anti-inflammatory and antimicrobial effects [53] and benzene compounds are known to contribute to systemic toxicity when absorbed through the cuticle [65].

The present SEM data showed that the in vitro exposure to the LC90 of *C. andromeda*’s venom caused body wall swelling and oedema along with cuticle deformation, such as destruction of stichosomes, formation of longitudinal creases, and the loss of cuticular striations, which are consistent with findings of Elmahy et al. [33]. In fact, all case reports on jellyfish stings showed that the envenomated area frequently experienced muscle swelling, an increase in lactic acid formation, haemorrhages, and stimulated inflammation in muscle tissue [66, 67]. Furthermore [61], detected ultrastructural damage, swelling, loss of annulations, formation of depression zones, sloughing and cracking as effects of using the sea cucumber *Holothuria polii* against *T. spiralis*.

The cuticular alterations after *C. andromeda’s* venom exposure could be related to its proteinaceous porins content [64] and benzene compounds [65] which contribute to perforations in the cellular membranes and systemic toxicity when absorbed through the cuticle. On the other hand, the ultrastructure alterations after ABZ treatment are directly related to its attachment to cuticular tubulin, which compromises the parasite’s structural integrity [68].

The significantly higher weight gain observed during the acute exposure phase (first 7 days) compared to the chronic phase (36 days) may be attributed to several physiological and experimental factors. During the acute phase, mice were initially adapting to the treatment, which might have stimulated appetite or altered metabolism temporarily, resulting in a relatively rapid weight gain. This initial gain could also reflect compensatory growth or a stress-related hyperphagic response. As the exposure continued into the chronic phase, the mice may have reached a state of metabolic adaptation, leading to a stabilization of weight gain over time [69].

Liver enzyme metabolism, gut microbiota effects, rapid absorption, detoxification, and excretion are challenges for in vivo pharmaceutical applications of the alternative anthelminthic [70]. The current in vivo assay investigated the possible alterations following venom administration on gut pathophysiology and histology, blood parameters, and liver function of the infected and treated mice.

Significant hematological alterations have been observed in *T. spiralis* infected mice [71]. The increase in neutrophil and lymphocyte counts, which are early markers of the host’s immune response to the parasitic infection, was previously noticed in trichinosis patients [72]. The present results showed a significant increase in the total WBC count in the infected untreated group compared with the control group.

Contrariwise, red blood cell (RBC) counts, hematocrit (Hct) values and hemoglobin (Hb) concentration tend to decrease during microbial infection, witnessing a mild anemia. Additionally, the significant reduction in platelet (PLT) counts as well as the thrombocyte levels after *T. spiralis* infection may be attributed to the mechanically and enzymatically damage of vascular endothelium through larval migration, which leads to localized bleeding. Endothelial disruption exposes subendothelial collagen and matrix components, triggering platelet activation, and aggregation to form micro-clot, limiting blood loss and initiating vascular repair [73].

In the present study, the levels of RBCs, Hb %, HCT % and PLT were decreased significantly in the infected untreated group compared to the control group. After treatment with *C. andromeda*’s venom, the levels of these parameters were significantly increased compared with the infected untreated group, which may reflect the efficacy of the venom in reducing the number of adult worms and larvae and improving the mice’s appetite and overall health.

These hematological alterations reflect the host’s immune response and the pathophysiological impact of *T. spiralis* infection, highlighting the importance of monitoring these parameters for early diagnosis and effective management of Trichinellosis.

Previous studies have reported the involvement of *T. spiralis* in significant increases in serum ALT and AST, suggesting possible injury to the liver and muscle during the parasitic invasion [74, 75]. Stewart [76] indicated that during the period from Days 6 to 18 PI (acute phase) in mice afflicted with *Trichinella*, ALT level increased then started to decrease in the chronic phase, correlating with the advancement of the acute phase of the parasite, the onset of gut inflammation, and the invasion of cardiac and voluntary muscle fibers by migrating *T. spiralis* larvae. The present data agree with this observation. Additionally, the data show that serum ALT and AST levels during both the acute and chronic phases are increased compared with the uninfected control group. Both treated groups exhibited a significant decrease in enzyme levels compared to the infected untreated group. Elevated levels of these enzymes may arise from the direct invasion of liver cells, triggering an immunological reaction by releasing interleukins or toxins by the parasite during its migration route.

Low levels of serum albumin and globulin may indicate liver or kidney disorders, nutritional deficiencies, prolonged bleeding and anemia [71]. The decrease in serum total protein and albumin in *T. spiralis*-infected mice may result from reduced dietary amino acids and impaired absorption [77]. The marked reduction in total protein levels in the current work was noted in the infected untreated group compared to the control group, while all treated groups showed an increase in levels relative to the infected untreated group. The alignment between liver enzyme results and total protein levels indicates liver injury related to trichinosis.

Antioxidants, as significant suppressors of reactive oxygen species (ROS) and free radicals generated by parasitic activities [78], inhibit lipid peroxidation and improve the host immune response [2]. The parasite’s modulation of ROS production, and consequently the subsequent oxidative stress, is considered the main factor of the pathogenic impact of *Trichinella*. SOD, CAT, and GSH, as important antioxidant factors, promote cellular health by neutralizing ROS, as well as protecting proteins, lipids, and DNA from damage [79–81]. Additionally, the elevation of the MDA level indicates the oxidative stress and lipid peroxidation that accompany trichinosis [82]. An increase in antioxidant activity during trichinellosis may be due to *T. spiralis* larvae prompting phagocytes to produce free radicals. Therefore, SOD and GPx levels in the blood remained elevated for at least nine weeks post-infection, helping to protect the host from self-damage and the harmful effects of free radicals from *Trichinella* larvae [83]. The present results, which are supported by [61], indicated a reduction of SOD, CAT, and GSH levels in the infected untreated group, alongside a significant increase in the MDA level when compared to the control group. However, the infected groups that received either ABZ or venom showed an increase in SOD, CAT, and GSH and a decrease in MDA levels when compared to the infected untreated group.

The role of IL-6 as a pro-inflammatory cytokine is essential in improving host defence against pathogens and in chronic inflammation [84, 85]. It is well known that in trichinosis, the worms release compounds in their excretory-secretory (ES) secretions, which interact with antigen-presenting cells, encouraging Th2-biased immune responses [86]. In contrast to El-Saidy et al. [61], the current work detected higher levels of IL-6 in the infected untreated group compared with the control group, which decreased in both infected treated groups with ABZ or venom. These results may indicate that the host probably had a mixed Th1/Th2 immune inflammatory response in the adult phase of *T. spiralis* [87].

In accordance with El-kady et al. [88], the present findings demonstrated that the small intestine undergoes significant alterations during the acute phase of trichinosis. These alterations include atrophy, shortening of the villi, hyperplasia of the lining epithelia, and destruction and disarray of the villi. Moreover, there is a dense inflammatory infiltrate within the lamina propria, primarily made up of neutrophils, lymphocytes, and macrophages; dysplastic mucosal glands (crypts) with hyperchromatic epithelial cells (pyknotic nuclei); and an increase in goblet cells. An increased level of IL-5 dependent eosinophils has been previously reported to expel adult worms and eliminate newborn larvae of *T. spiralis* [89, 90].

The current increase in goblet cell counts in the intestine of all infected mice groups compared to uninfected mice reflects its significant role in the secretion of mucins and in the formation of a protective viscous barrier against invading intestinal nematodes [91]. The administration of venom and albendazole to *T. spiralis*-infected mice exhibited an improvement in the small intestine architecture as proven by regular tall villi with normal lining epithelia, normal goblet cells, and well-organized mucosal glands (crypts). These results were consistent with those of El-kady et al. [88], who reported that the administration of A. annua and albendazole to *T. spiralis*-infected mice restored the normal villi structure and reduced inflammatory cellular infiltration.

Additionally, the current histopathological changes of the skeletal muscles of the infected group showed disorganization of the muscular tissue, disarrangement of the myocytes, and numerous larvae embedded in the muscles within intact capsules that are surrounded by dense inflammatory infiltrates. These results were similar to a study by Bruschi and Chiumiento [92], who documented that *T. spiralis* mechanically damages the skeletal muscle cells and recruits inflammatory cells via the production of free radicals. Sections of skeletal muscles of the infected group treated with albendazole exhibited improvement of the muscular tissue, as represented by necrosis of larvae, disruption of the capsule, and penetration of inflammatory cells within the capsule. These results are in accordance with Khedr et al. [93], who reported that albendazole was effective and its combination with Cur-NE showed significant potentiation against adult worms and muscle larvae and alleviated the pathology in both acute and chronic models.

In the current study, a significant decrease in the inflammatory cellular infiltration and goblet cell count in the treated groups coincided with the lower adult worm count. Likewise, the restoration of the muscle fibers’ natural appearance and a notable decrease in the infiltration of inflammatory cells occurred in the treated groups once the larval count in the muscles was reduced. The presence of inflammatory cells that generate large quantities of reactive oxygen species and various stress markers contributes to host tissue damage during the muscle phase of trichinellosis, in addition to the invasive parasite itself [10].

Mature *T. spiralis* worms cause acute inflammatory reactions that lead to several intestinal pathological alterations [94–96]. The current study detected that *T. spiralis* led to a significant increase in expression of P53 by immunohistochemistry and a high cytoplasmic staining intensity in the enterocytes of small intestine sections. These findings align with the report by Khan and Collins [8] and Karmańska et al. [97], which detected that *T. spiralis* triggers apoptosis and influences certain cells involved in inflammatory infiltration within the lamina propria of the jejunal mucosa, as well as in the striated muscle of infected mice. Furthermore, Kazura and Aikawa [98] showed that the nematode *Toxocara canis* infection led to a significant increase in caspase-3 expression in the brains of control mice, regardless of whether the infection was in the early or late stages. Additionally, our findings indicate that treatment with albendazole and venom weakened the expression and led to the loss of P53 immunostaining in sections of the small intestine. These results are previously reported by [98], who recorded that the administration of artemether and albendazole treatments weakened the expression of caspase-3 in a *Toxocara* model. Artemether also outperformed albendazole at both time points post-infection in reducing caspase-3 expression.

Although P53 is not required for the activation of apoptosis in the basophilic cytoplasm, it may contribute to mitochondria-mediated apoptosis there. There are two ways that parasites might cause apoptosis: directly through active mediators or indirectly through inflammatory mediators. In many cases, parasite infections result in tissue damage due to apoptosis, a type of host-cell death. The increased production of many stress markers indicates that the oxidative stress state caused by *Trichinella* infection is one of the primary causes of this damage [99].

The current results demonstrated that P53 was expressed as a markedly intense immunostaining in the sarcoplasm of myocytes and cellular infiltrates surrounding larvae in the skeletal muscles. *T. spiralis* induced apoptosis and necrosis in myocytes through the histological results of skeletal muscles with increased pyknotic nuclei and degeneration of myocytes with a marked cytoplasmic vacuolation. In accordance, Bakr et al. [100] demonstrated that *T. spiralis* encysts as larvae that are surrounded by a collagen capsule and mild inflammatory cellular infiltration in rat skeletal muscle fibers; in addition to a significant elevation of the apoptotic protein p53 and significant depletion in Bcl2, as well as muscle injury. Also, Karmańska et al. [97] reported that *T. spiralis* induces apoptosis which affects some cells of the inflammatory infiltration in the lamina propria of the jejunal mucosa and in striated muscle of mice infected with *T. spiralis*. These results are in agreement with Ibrahim et al. [101] who reported that rats infected with *T. spiralis* exhibited degenerative alterations along with noticeable apoptosis. The treated groups with albendazole and venom showed improvement and an obvious disappearance of P53 immunostain expression in the sarcoplasm of myocytes of skeletal muscles.

In our study, the mean area percent was used to quantitatively assess the extent of p53 activation in response to venom. An increase in the mean area percent of the infected group (G II) indicates upregulation of p53 expression, suggesting activation of apoptotic pathways, often as a defense mechanism against cellular damage or toxicity. Conversely, a reduction in p53 expression in the infected and venom**-**treated group (G V) may imply an impaired cellular response or potential evasion of apoptotic pathways. Therefore, this measurement provides insight into the molecular and cellular impact of the treatment, helping to correlate histological findings with underlying biological mechanisms.

## Conclusion

The current study demonstrates that *Cassiopea andromeda* venom exhibits potent anthelmintic, hepatoprotective, and immunomodulatory properties against *Trichinella spiralis* infection in murine models. Venom administration notably decreased parasite burdens, enhanced hepatic and intestinal tissue histology, and normalized hematological and antioxidant parameters. Additionally, the observed downregulation of p53 expression implies a reduction in tissue apoptosis and inflammation. These findings highlight that venom could serve as a promising treatment for *T. spiralis* infection and may be a viable alternative to albendazole for treating trichinellosis.

Further research should explore *C. andromeda*’s venom for modulating host immunity and preventing *T. spiralis*-induced histopathological changes. There is an urgent need to investigate marine venoms as safe, effective nematocidal agents with therapeutic potential. Peptide venoms from marine sources require extensive in vivo toxicological studies, including structural, chemical, recombinant production, and target identification.

## Supplementary Information

Below is the link to the electronic supplementary material.

Supplementary Material 1

## Data Availability

Not applicable.
