# Supplementary material for: Marine Biotoxins as Potential Nematocide: Impact of Cassiopea andromeda Venom Against Acute and Chronic Trichinellosis in Murine model
Source: Acta Parasitol. 2026 Apr 6;71(2):80. doi: 10.1007/s11686-026-01256-7 (PMC13053410; doi:10.1007/s11686-026-01256-7)
Supplement: Supplementary file 1 — Supplementary Material 1 [file 11686_2026_1256_MOESM1_ESM.pdf]

CERTIFICATE OF ENGLISH EDITING

This certificate confirms that the manuscript listed below was edited by an expert English editor with a PhD.

The following issues were corrected: grammar, spelling, punctuation, sentence structure, and phrasing.

Manuscript title

Marine Biotoxins as Potential Nematocide: Impact of Cassiopea andromeda Venom Against Acute and Chronic Trichinellosis in Murine model

Date issued

21 September 2025

Certificate Number

A35/2025

Cairo, Egypt

SCIENTIFIC SOLUTIONS

M. A. Ali
